# A novel mode of control of nickel uptake by a multifunctional metallochaperone

**DOI:** 10.1371/journal.ppat.1009193

**Published:** 2021-01-14

**Authors:** Milica Denic, Evelyne Turlin, Valérie Michel, Frédéric Fischer, Mozhgan Khorasani-Motlagh, Deborah Zamble, Daniel Vinella, Hilde de Reuse

**Affiliations:** 1 Institut Pasteur, Département de Microbiologie, Unité Pathogenèse de *Helicobacter*, CNRS UMR 2001, Paris, France; 2 Université de Paris, Sorbonne Paris Cité, Cellule Pasteur, Paris, France; 3 Génétique Moléculaire, Génomique, Microbiologie, UMR 7156, CNRS, Université de Strasbourg, Institut de Botanique, Strasbourg, France; 4 Department of Chemistry, University of Toronto, Toronto, Ontario, Canada; 5 Department of Biochemistry, University of Toronto, Toronto, Ontario, Canada; University of Illinois, UNITED STATES

## Abstract

Cellular metal homeostasis is a critical process for all organisms, requiring tight regulation. In the major pathogen *Helicobacter pylori*, the acquisition of nickel is an essential virulence determinant as this metal is a cofactor for the acid-resistance enzyme, urease. Nickel uptake relies on the NixA permease and the NiuBDE ABC transporter. Till now, bacterial metal transporters were reported to be controlled at their transcriptional level. Here we uncovered post-translational regulation of the essential Niu transporter in *H*. *pylori*. Indeed, we demonstrate that SlyD, a protein combining peptidyl-prolyl isomerase (PPIase), chaperone, and metal-binding properties, is required for the activity of the Niu transporter. Using two-hybrid assays, we found that SlyD directly interacts with the NiuD permease subunit and identified a motif critical for this contact. Mutants of the different SlyD functional domains were constructed and used to perform *in vitro* PPIase activity assays and four different *in vivo* tests measuring nickel intracellular accumulation or transport in *H*. *pylori*. *In vitro*, SlyD PPIase activity is down-regulated by nickel, independently of its C-terminal region reported to bind metals. *In vivo*, a role of SlyD PPIase function was only revealed upon exposure to high nickel concentrations. Most importantly, the IF chaperone domain of SlyD was shown to be mandatory for Niu activation under all *in vivo* conditions. These data suggest that SlyD is required for the active functional conformation of the Niu permease and regulates its activity through a novel mechanism implying direct protein interaction, thereby acting as a gatekeeper of nickel uptake. Finally, in agreement with a central role of SlyD, this protein is essential for the colonization of the mouse model by *H*. *pylori*.

## Author summary

Metal ions are essential for the viability of all living organisms. Indeed, more than one-third of all proteins need metal cofactors for their function. Intracellular metal concentrations require tight control as non-physiological amounts are very toxic. In particular, nickel plays a unique role in *Helicobacter pylori*, a bacterial pathogen that colonizes the stomach of about half of the human population worldwide and is associated with the development of gastric cancer. Nickel is essential for *H*. *pylori* as it is the cofactor of urease, an enzyme indispensable for resistance to the gastric acidity of the stomach and thus for *in vivo* colonization. To import nickel despite its scarcity in the human body, *H*. *pylori* requires efficient uptake mechanisms. Till now, control of nickel uptake was only reported to rely on transcriptional regulators. In the present study, we uncovered a novel mechanism of regulation of nickel acquisition. SlyD, a multifunctional enzyme was found to control, by direct protein interaction, the activity of an essential nickel uptake system in *H*. *pylori*. We revealed that the SlyD chaperone activity is mandatory for the active conformation and thus functionality of the nickel permease.

## Introduction

Metal ions are essential for the viability of all living organisms. Metals are known to be involved in over 40% of enzymatic reactions, and metal-binding proteins carry out at least one step in almost all biological pathways, notably in essential processes such as metabolism, respiration and photosynthesis [1]. In cells, the amount and distribution of each metal must be finely tuned, to prevent toxic effects of some metal ions and to ensure that metalloproteins bind their cognate metal ion, thereby preventing mis-metalation [2].

The allocation of transition metal ions has also been associated with bacterial virulence [3]. To protect themselves, the infected hosts combat bacterial colonization by limiting the bioavailability of metal ions, a process known as nutritional immunity [4]. Conversely, the hosts are well known to exploit the potential toxicity of metal ions to intoxicate invading pathogens [5]. To counter the host protective strategies and maintain proper cytoplasmic metal ion abundance, pathogenic bacteria have evolved a network of metalloregulatory processes that control import and efflux, as well as storage and intracellular trafficking. Many efforts have gone into understanding iron homeostasis and trafficking. Less information is known for nickel although it is a cofactor of at least nine enzymes important for metabolism or virulence [6,7]. We report here an original mechanism by which *Helicobacter pylori* controls nickel import.

The bacterium *H*. *pylori* is a pathogen that infects the stomach of about half of the human population and is associated with the development of gastritis, peptic ulcer disease and adenocarcinoma causing the death of approximately 800,000 people each year in the world [8]. *H*. *pylori* is a model bacterium for the study of metal metabolism because its survival depends on two nickel enzymes, urease and [NiFe]-hydrogenase, both essential for colonization of the stomach and important for the virulence of the bacterium [9]. Urease catalyzes the hydrolysis of urea to ammonium, which serves as a buffer that allows *H*. *pylori* to survive the acidity of the stomach. Urease represents about 6% of the soluble proteins of *H*. *pylori* and contains 24 nickel ions per active complex [10]. The [NiFe]-hydrogenase is also essential for colonization by allowing the bacterium to utilize molecular hydrogen as an energy substrate [11]. Therefore, *H*. *pylori* needs a constant and significant flow of nickel to ensure its survival, but in the host stomach, the nickel concentration is very low (about 0.5 nM). Accordingly, we previously observed that *H*. *pylori* and other gastric *Helicobacter* species have, over the course of evolution, acquired several genes that encode factors involved in the transport and the storage of nickel, highlighting the importance of nickel import in the adaptation of bacteria to the hostile environment of the stomach [12,13].

In *H*. *pylori*, the import of nickel through the outer membrane is carried out by the FrpB4 TonB-dependent transporter (TBDT) [14]. The metal is then transported into the cytoplasm by one of the two sole nickel uptake systems of *H*. *pylori*, NixA and Niu [13,15]. The NixA permease is a member of the NiCoT family that uses the physicochemical gradient of the cytoplasmic membrane as an energy source. The NiuBDE (in short Niu) ABC transporter energized by NTP has recently been identified in our team [13,16]. For now, the only regulation of nickel uptake that was reported in *H*. *pylori* relies on transcriptional repression by NikR, a pleiotropic nickel responsive regulator [17,18]. Both nickel transporters are required for efficient colonization of the mouse model, with Niu even being essential for the process [13]. To date, only one pathway of nickel export has been reported in *H*. *pylori*, the proton-driven metal efflux pump, *CznABC* [19]. *H*. *pylori* also expresses several unique proteins able to bind nickel in the cytoplasm. First, HspA the sole homologue of the chaperone protein GroES in *H*. *pylori*, has a C-terminal histidine and cysteine-rich extension, absent from non-*Helicobacter* bacteria that behaves like a nickel sequestration domain [20]. Second, *H*. *pylori* produces two small histidine-rich proteins, Hpn and Hpn-2 [21–23]. HspA and Hpn/Hpn-2 contribute to nickel storage but also to the control of hydrogenase and urease activity, respectively [12,20]. One other nickel-binding protein in the *H*. *pylori* cytoplasm is SlyD [24], but the physiological function of this protein was not defined and was investigated here.

SlyD is a member of the FK506-binding protein (FKBP) family with peptidyl-prolyl *cis-trans* isomerase activity (PPIase) [25,26]. These enzymes catalyze the *cis*/*trans*-isomerization of peptidyl-prolyl (XAA-Pro) bonds which can occur spontaneously but is often a rate-limiting step for protein folding, thus modulating protein activity, interaction with protein partners or other protein signaling [27,28]. SlyD belongs to a subfamily of PPIases that is found in bacteria and archaea, characterized by the insertion of a chaperone domain into the FKBP domain "insert-in-flap (IF)" which enables it to also function as an efficient molecular chaperone, thus preventing protein aggregation [29,30]. Although distinct, the IF and the FKBP domains are mechanistically linked [31,32]. Furthermore, bacterial SlyD homologues have a C-terminal extension varying from 2 to 50 residues in length [24,33] which binds divalent cations. In *E*. *coli*, the extension contains 13 histidine and 6 cysteine residues, along with multiple carboxylate amino acids, and can bind a variety of divalent cations including up to 7 nickel ions per SlyD molecule [25,34]. Notably, nickel binding to the C-terminal region was shown to down regulate the *E*. *coli* SlyD PPIase activity [25,35]. The N-terminal domain was also found to harbor an additional nickel binding site [33].

The SlyD protein was first studied in *E*. *coli* where it was characterized as a host factor during bacteriophage ΦX174 infection, stabilizing the viral lysis protein E [36]. However, the extent of the cellular function and the physiological substrates of SlyD are still not clear. It has been shown that the chaperone IF domain can bind the TAT (Twin-Arginine Translocation) secretion signal sequences, facilitating the translocation of folded proteins from the cytoplasm to the periplasm [37]. *E*. *coli* SlyD also plays a role in the maturation of the [NiFe]-hydrogenase and *slyD* mutant strains display two-to-ten times lower [NiFe]-hydrogenase activity compared to that of the wild-type bacteria [38,39]. An *in vitro* interaction between the SlyD IF domain and the HypB hydrogenase accessory protein appears to be required for nickel insertion and maturation of the hydrogenase complex, a process that depends on the C-terminal nickel-binding region [40–42].

Much less is known about the role of SlyD protein in *H*. *pylori*. The structure of its C-terminus truncated form in solution has been determined [24]. The *H*. *pylori* SlyD C-terminal extension contains multiple metal-binding residues (5 histidines and 5 cysteines), allowing the purified protein to bind divalent cations, such as particularly nickel [24]. Previous interactomic studies suggested that SlyD is part of a complex comprising the UreA urease subunit and the hydrogenase maturation accessory protein HypB [43,44]. It was reported later that *H*. *pylori* SlyD binds, through its IF domain, to HypB and to the TAT signal peptide of the periplasmic HydA [NiFe]-hydrogenase subunit [24,45]. Additionally, it has been shown that the concomitant deletion of *slyD* and *hypA* resulted in diminished urease activity [46]. However, the actual role of SlyD in urease activity in this context is not fully understood.

In the present study, we explored for the first time the role of the SlyD protein in nickel metabolism and virulence of *H*. *pylori* and demonstrated that SlyD is required for colonization of a mouse model by *H*. *pylori*. By combining assays to measure metal resistance, nickel transport and accumulation, we provide evidence that SlyD controls nickel uptake in *H*. *pylori*. In addition, we show that SlyD directly interacts with the NiuD permease subunit of the NiuBDE ABC transporter. Altogether, the results indicate that SlyD performs an essential role in *H*. *pylori*, and support a model of a novel mode of regulation of nickel acquisition.

## Results

### Mutagenesis of the *Helicobacter pylori* cytoplasmic SlyD protein

**Fig 1:**
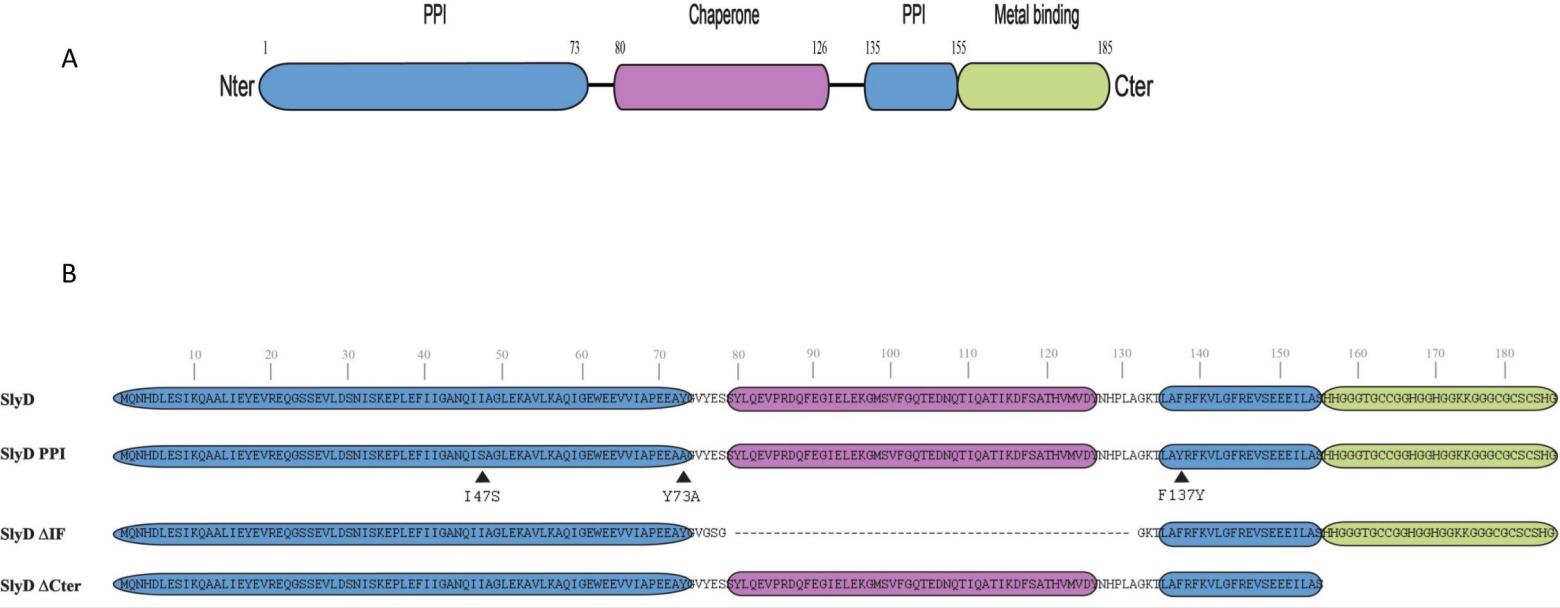
Illustration of the *H*. *pylori* SlyD wild type and mutant proteins. **A.** Schematic representation of the functional domains of the SlyD protein of *H*. *pylori* strain B128, with indication of the corresponding encompassing residues. The regions required for the peptidyl-prolyl isomerase activity (PPIase) are colored in blue, the "inserted in Flap" IF chaperone domain is colored in pink and the C-terminal metal-binding region is colored in green. **B.** Illustration of the different SlyD mutants, SlyD-PPI, SlyD-ΔIF and SlyD-ΔCter. The three residues (I47S, Y73A and F137Y) changed in the SlyD-PPI mutant are marked by a black arrow.

To study the role of SlyD in *H*. *pylori*, we first constructed a mutant with a complete deletion of the *slyD* open reading frame in strain B128 [47,48] and a complemented strain in which the wild type *slyD* gene was reintroduced at the native locus in the *ΔslyD* mutant. To dissect the contributions of the different activities of the SlyD protein, a series of strains expressing mutant versions of SlyD from the native chromosomal locus under the control of the WT promoter was also constructed (Fig 1). Structural modeling based on the NMR structures of the SlyD proteins from *E*. *coli* [49] and from *H*. *pylori* strain 26695 [24] along with analysis of conserved residues in the *H*. *pylori* SlyD protein (S1A Fig), allowed us to predict the locations of the functional domains of SlyD (Figs 1A and S1B). The mutants are represented in Fig 1B. The first mutant designated SlyD-PPI, carries substitutions of three residues that are predicted to take part in the peptidyl-prolyl isomerase activity, I47S, Y73A and F137Y. The second mutant, designated SlyD-ΔIF carries a deletion of 56 residues encompassing the IF chaperone domain. In the last mutant strain, designated SlyD-ΔCter, the C-terminal His and Cys region-rich starting from residue 155 was removed. The strains expressing the SlyD mutants were viable and presented negligible growth and viability defects (S2A Fig).

**Fig 2:**
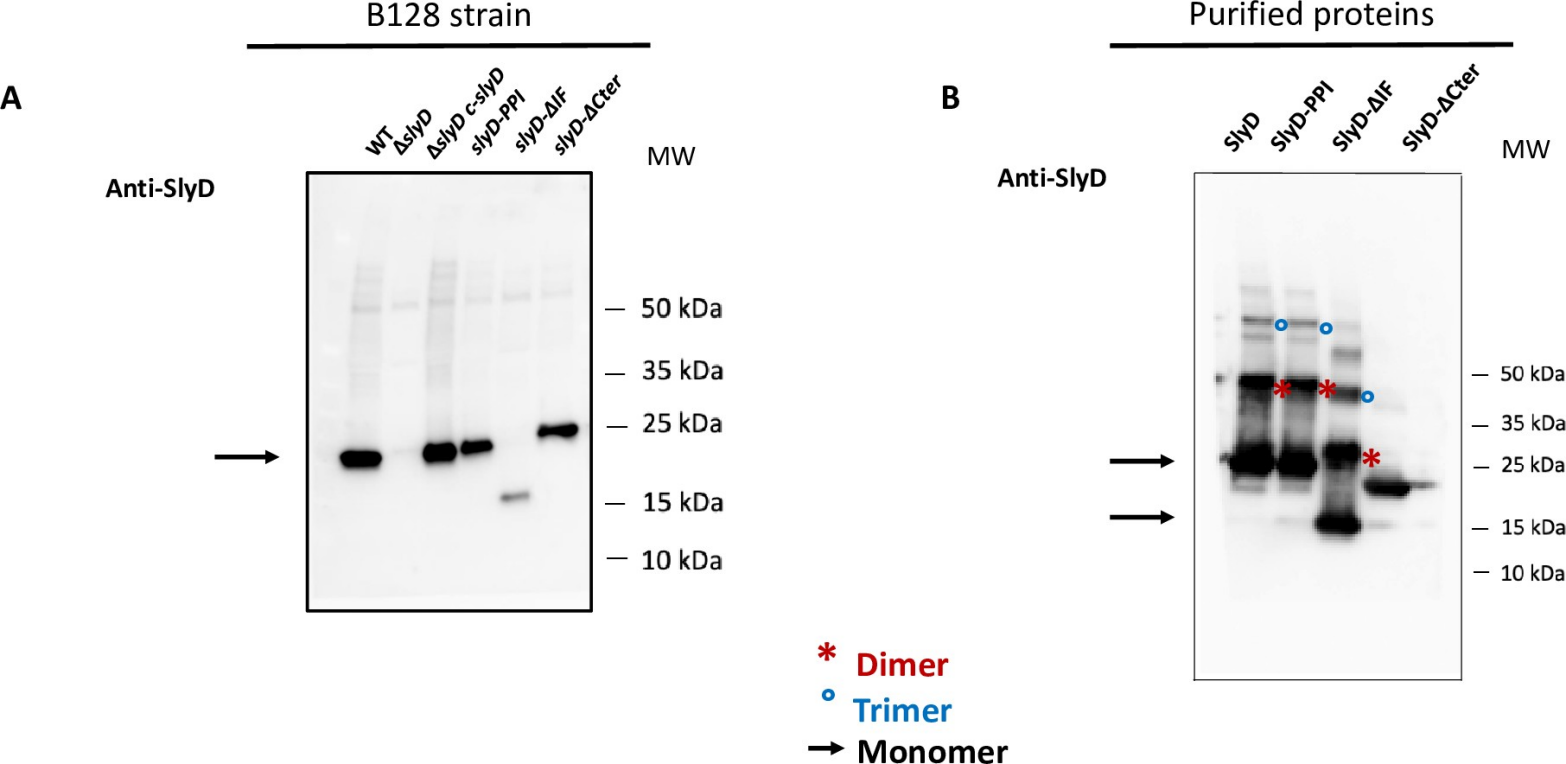
Analysis of the production of SlyD wild type and mutant proteins in *H*. *pylori*. **A.** Western blot of equal amounts of total extracts, under reducing conditions, from *H*. *pylori* B128 WT strain and B128-derived mutants carrying the following mutations *ΔslyD*, *ΔslyD c-slyD* (complemented mutant), *slyD-PPI*, *slyD-ΔIF*, and *slyD-ΔCter* strain, that were probed with specific anti-SlyD polyclonal antibodies prepared during this study. An arrow shows the position of the SlyD protein. **B.** Western blot of purified recombinant SlyD proteins (WT, SlyD-PPI, SlyD-ΔIF and SlyD-ΔCter) probed with specific anti-SlyD polyclonal antibodies. An arrow shows the position of the monomeric SlyD proteins, red stars and blue circles highlight SlyD dimers and trimers, respectively.

To evaluate the expression of each SlyD construct in *H*. *pylori*, we produced specific anti-SlyD antibodies and performed Western analysis under reducing conditions (with DTT), revealing production of all SlyD variants, although at lower levels for the SlyD-ΔIF mutants (Fig 2A). The subcellular localization of SlyD was also analyzed by using a validated *H*. *pylori* fractionation procedure (S2B Fig). We found that SlyD protein is exclusively present in the soluble fraction strongly suggesting that it is a cytoplasmic protein.

### Peptidyl-prolyl isomerase (PPIase) activity and nickel regulation of the *H*. *pylori* SlyD protein

To investigate the biochemical activities of the *H*. *pylori* SlyD variants, the corresponding recombinant proteins were expressed and purified from *E*. *coli*. Under non-reducing conditions, we observed that the SlyD proteins form multimers (probably dimers and trimers) that are not observed upon analysis of the SlyD-ΔCter mutant (Fig 2B) or under reducing conditions (Fig 2A). These forms likely result from the spontaneous formation of disulfide bounds between the cysteine residues present in the C-terminal domain of SlyD (Fig 1B).

**Fig 3:**
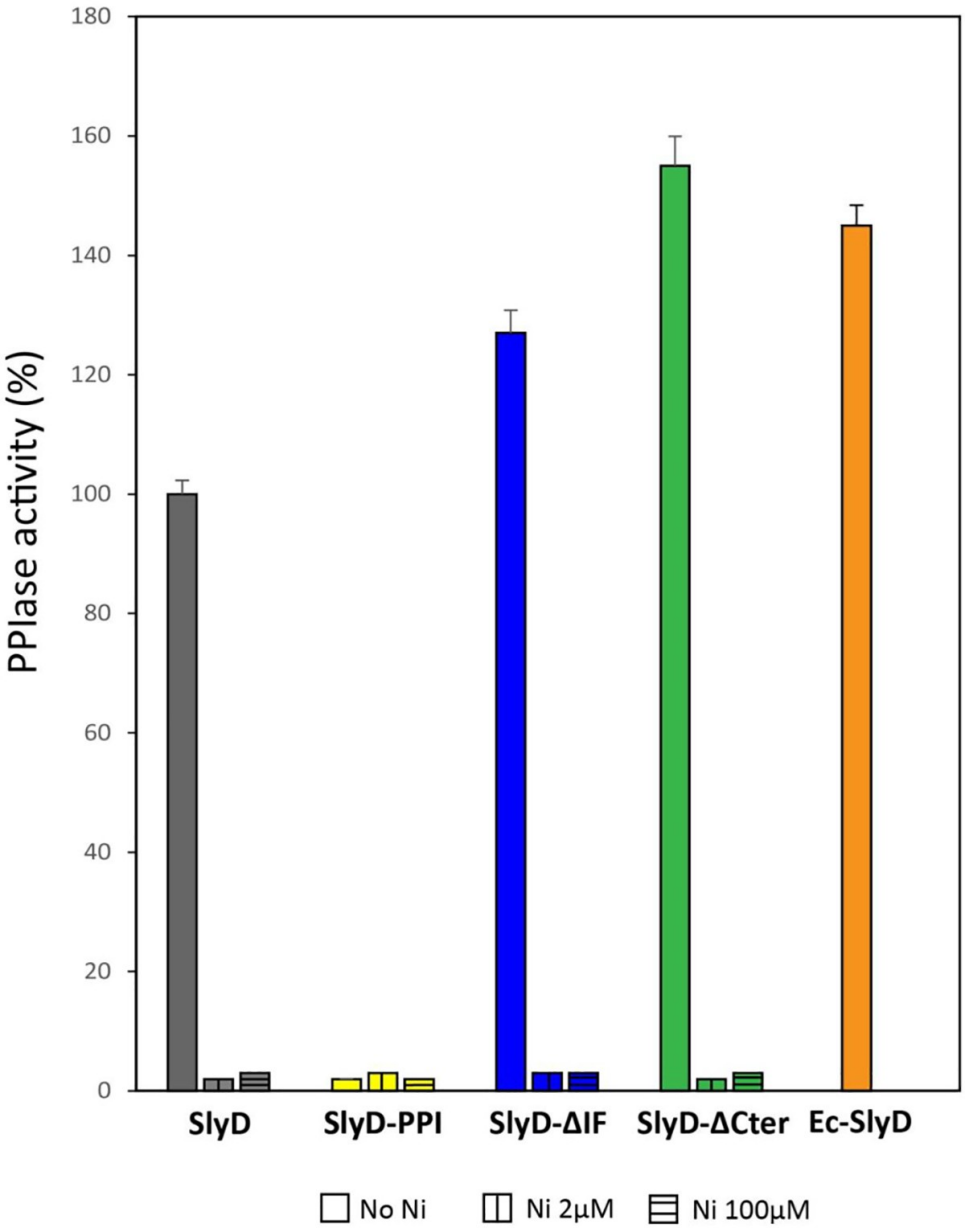
*In vitro* PPIase activity and nickel regulation of *H*. *pylori* wild type and mutant SlyD proteins. PPIase activities of WT and mutant SlyD proteins, measured without or with 2 μM or 100 μM NiSO_4_. The PPIase activity of purified *E*. *coli* SlyD (*Ec*SlyD) is also presented as a control. The data (S3B Fig) were fit to second-order rate equations and the PPIase activities are expressed as a percentage of the activity of the wild type *H*. *pylori* SlyD protein. Grey bars correspond to the wild type protein, yellow to the SlyD-PPI mutant, blue to the SlyD-ΔIF mutant and green to the SlyD-ΔCter mutant. The orange bar corresponds to the *E*. *coli* SlyD protein. The values are the averages from three replicates and error bars represent the standard deviation.

Circular dichroism spectroscopy analysis of the different SlyD proteins showed that the mutations did not result in major secondary structure changes (S3A Fig). Next, the PPIase activities of the purified wild type and mutant SlyD proteins were assayed by using an *in vitro* assay in which *cis* to *trans* prolyl isomerization of a tetrapeptide substrate is followed by monitoring the electronic absorption at 314 nm [50]. Purified *E*. *coli* SlyD protein served as a positive control (Fig 3). Our measurements revealed that the PPIase activity of wild type *H*. *pylori* SlyD protein is similar (84%) to that of the *E*. *coli* protein. The *H*. *pylori* SlyD-ΔIF and SlyD-ΔCter mutants retained even higher activity than that of the WT protein (145% and 150%, respectively) (Fig 3). In contrast the SlyD-PPI mutant had, as anticipated, completely lost detectable PPIase activity.

*In vitro* PPIase activity of *E*. *coli* SlyD was reported to be negatively affected upon nickel binding [25]. Purified *H*. *pylori* SlyD protein was previously reported to bind nickel ions with a *K*_*d*_ value of 2.74 ± 0.26 μM [24]. Therefore, the PPIase activities of purified SlyD proteins (2 μM) were assayed in the presence of 2 μM or 100 μM NiSO_4_. It was found that both nickel concentrations totally inhibit the PPIase activity of all the SlyD variants (Figs 3 and S3B–S3C).

These data established the PPIase activity of the *H*. *pylori* SlyD protein and validate the importance of the three predicted active site residues. In addition, they demonstrate that neither the chaperone domain nor the C-terminal region is required for PPIase activity *in vitro* and that stoichiometric nickel inhibits this activity even in the absence of the C-terminal metal-binding region.

### SlyD inactivation increases *H*. *pylori* tolerance to high nickel concentrations

The nickel-dependent regulation of the SlyD PPIase activity, along with its established nickel-binding properties [24], prompted us to examine whether this protein plays a role in nickel metabolism, transport and/or trafficking in *H*. *pylori*. We first tested whether SlyD is required for the activation of the nickel-dependent enzymes of *H*. *pylori*, urease and hydrogenase. We found that urease and hydrogenase activities are not affected in the *ΔslyD* mutant as compared to those of the wild type strain (S1 Table). In the closely related bacterium *Campylobacter jejuni*, wild type hydrogenase activity was also measured in the *ΔslyD* mutant [51]. These results demonstrated that hydrogenase activity, in these two epsilon proteobacteria, does not require SlyD, in contrast to the situation in *E*. *coli* [41].

**Fig 4:**
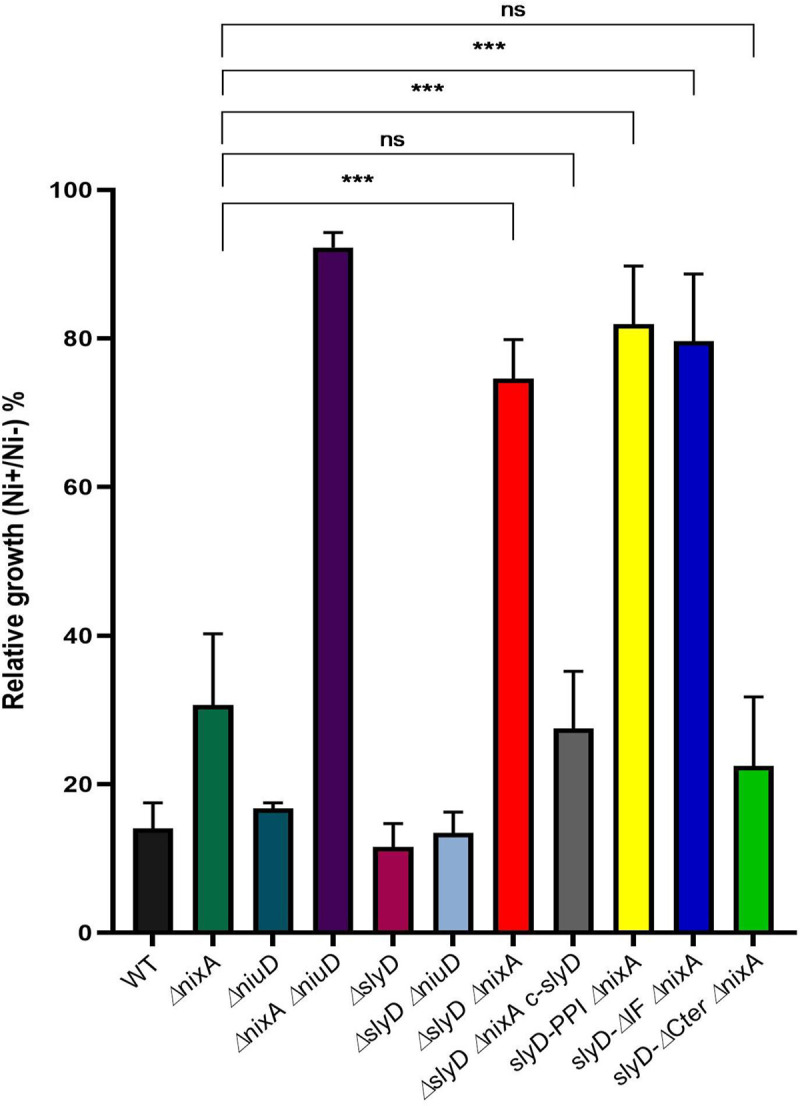
Tolerance of *H*. *pylori* wild type and mutant strains to nickel exposure. Growth of *H*. *pylori* B128 wild type strain, isogenic *slyD* mutants and a complemented strain (c-*slyD*) was measured after 24h in the presence of 1.5 mM NiCl_2_ or without added metal. The results are presented as the percentage of growth in the presence of nickel relative to growth in its absence. In this figure as well as in Figs 5, 6 and 7, the same color codes were used for the bars corresponding to each strain or mutant which name is indicated below each bar. Black bars correspond to the wild type strain, dark green to the *ΔnixA* mutant, dark blue to the *ΔniuD* mutant, violet to the Δ*nixA ΔniuD* mutant, dark red to the *ΔslyD* mutant, light blue to the *ΔslyD ΔniuD* mutant, bright red to *ΔslyD ΔnixA* mutant, light grey to the *ΔslyD ΔnixA* c-*slyD* mutant, yellow to the *slyD-PPI ΔnixA* mutant, bright blue to the *slyD-ΔIF ΔnixA* mutant and light green to the *slyD-ΔCter ΔnixA* mutant. The data correspond to the mean value of three independent experiments. Error bars represent the standard deviation. Statistics are presented only for the comparison with the *ΔnixA* mutant: *** corresponds to p<0.001 and "ns" for non-significant. S9 Fig presents the complete statistical analysis of these data.

Then, the tolerance of the wild type strain and *slyD* mutants to toxic nickel exposure was evaluated by measuring their growth at neutral pH in liquid BB medium after 24 h incubation with 1.5 mM NiCl_2_ (Fig 4). Enhanced tolerance might result from several mechanisms, among which decreased metal uptake, as we showed to be the case for an *H*. *pylori* mutant lacking nickel transporters [13].

Here, we first found that the *ΔslyD* mutant presented no difference in nickel tolerance. We next examined the effect of the *ΔslyD* mutation in combination with deletions of the genes encoding the nickel uptake systems, NixA or NiuD (Fig 4). The single and double *ΔnixA* and *ΔniuD* mutants behaved as we previously reported; the mutant deficient in both nickel transporters being strongly tolerant to nickel [13]. The nickel tolerance of the *ΔslyD ΔniuD* double mutant was similar to that of the single *ΔniuD* mutant. In contrast, it was found that the Δ*slyD* Δ*nixA* mutant was as highly tolerant to nickel as the *ΔnixA ΔniuD* mutant, suggesting that nickel uptake is strongly impaired in the *ΔslyD ΔnixA* strain. This phenotype could be complemented by the re-introduction of a wild type *slyD* copy (*c-slyD*), indeed the *ΔslyD ΔnixA c-slyD* strain recovered nickel tolerance levels comparable to that of the parental *ΔnixA* mutant (Fig 4). Then, the effect of mutations in the *slyD* gene was tested in combination with *ΔnixA* (Fig 4). Deletion of the C-terminal domain of SlyD (strain *slyD-*Δ*Cter* Δ*nixA*) did not impact the tolerance of bacteria to high nickel concentrations. However, the mutants carrying either the SlyD-PPI mutation (*slyD-PPI ΔnixA*) or the deletion of its chaperone domain (*slyD-ΔIF ΔnixA*) were insensitive to 1.5 mM nickel exposure just like the *ΔslyD ΔnixA* mutant, attesting of the lack of activity of *slyD* variants in this assay.

These results indicate that *H*. *pylori* SlyD plays an essential role in nickel transport and/or metabolism. In addition, under these test conditions, the C-terminal domain of SlyD is not essential for this activity but both the PPIase isomerase activity and chaperone domain of SlyD are required for its function related to nickel uptake or metabolism.

### SlyD inactivation results in reduced intracellular nickel availability

**Fig 5:**
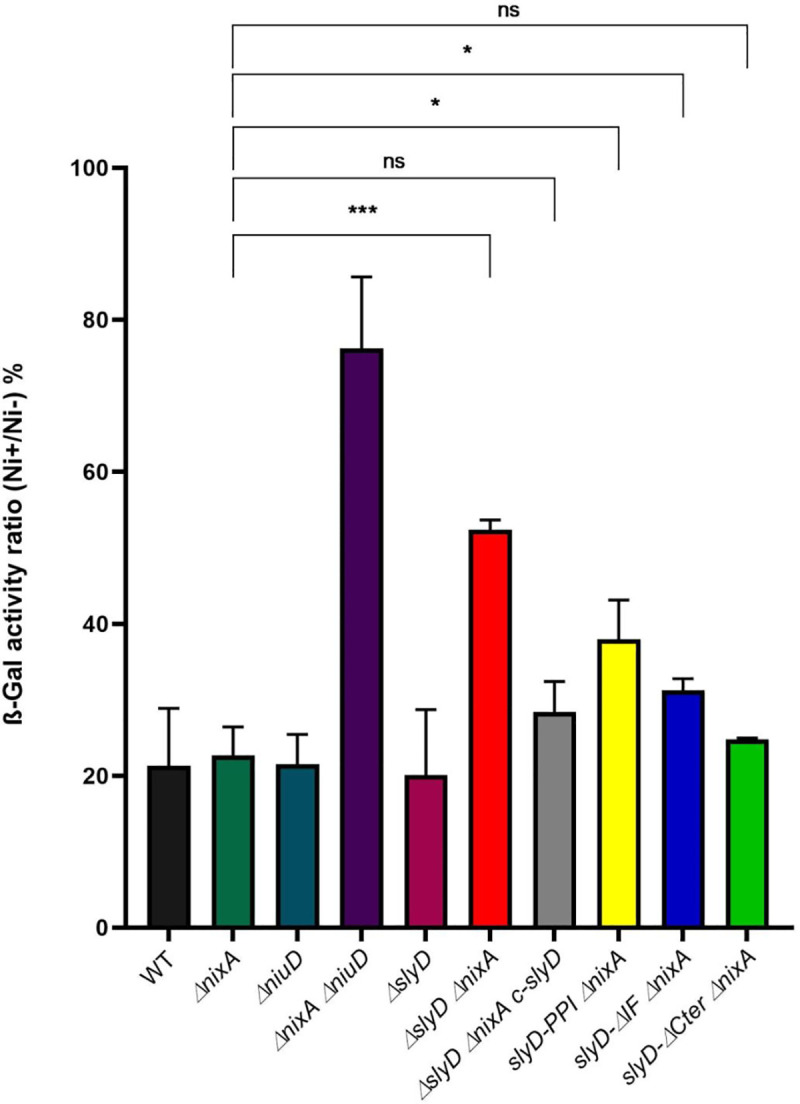
Evaluation of intracellular nickel availability of *H*. *pylori* wild type strain and isogenic mutants with a P*fecA3*::*lacZ* reporter fusion. ß-galactosidase activity of a P*fecA3*::*lacZ* reporter fusion expressed from a plasmid in different *H*. *pylori* B128-derived strains, after 24H exposure to 100 μM NiCl_2_. The expression of the fusion decreases in a NikR-dependent manner with increasing intracellular nickel concentration. In medium without added nickel, the ß-galactosidase activities of the different strains were found to be comparable (about 6,000 miller units). ß-galactosidase activities are presented as the ratio of activity measured in strains grown in the presence of 100 μM NiCl_2_ or in the absence of nickel supplementation, expressed as a percentage. Color codes of the bars are as in Fig 4. The data correspond to the mean value of three independent experiments (S5 Table). Error bars represent the standard deviation. Statistics are presented only for the comparison with the *ΔnixA* mutant. * corresponds to p<0.05, *** to p<0.001 and "ns" for non-significant. S9 Fig presents the complete statistical analysis of these data.

One possible explanation for these results is that SlyD modulates nickel accumulation into *H*. *pylori*. To test this hypothesis, we used an assay to indirectly evaluate the intracellular nickel content of different mutants by measuring the expression of *fecA3*, a gene known to be repressed by the transcriptional regulator NikR, in response to intracellular nickel concentrations [18]. First, we introduced in our mutant strains, a stable plasmid carrying a reporter gene fusion P*fecA3*::*lacZ* that we previously validated as a reporter of intracellular nickel bioavailability [13]. Fig 5 presents the ratio of ß-galactosidase activities of strains grown for 24 h in media supplemented with 100 μM NiCl_2_ versus without additional nickel. Lower ratios indicate stronger P*fecA3*::*lacZ* repression and thus higher intracellular nickel availability. The wild type strain, as well as the *ΔslyD* and *ΔnixA* individual mutants present ratios of about 15–20%, attesting of proper nickel-dependent repression and therefore efficient nickel uptake whereas the Δ*niuD* Δ*nixA* mutant presents a ratio of 70% as expected from its inability to import nickel [13]. The *ΔslyD ΔnixA* double mutant presents a ratio of 50% that is significantly different from that of the single *nixA* mutant, and could be complemented by the reintroduction of a wild type *slyD* gene. The phenotype of the three targeted *slyD* mutants in combination with *ΔnixA* was next analyzed. The *slyD-PPI ΔnixA* and *slyD*-*ΔIF ΔnixA* mutants presented a weak reduction in the repression by nickel while the deletion of the SlyD C-terminal domain did not prevent its activity. In conclusion, these results revealed an essential contribution of SlyD to the accumulation of intracellular nickel and demonstrated that the C-terminal domain is not required for this activity.

### Reduced intracellular nickel content of a *ΔslyD ΔnixA* mutant

**Fig 6:**
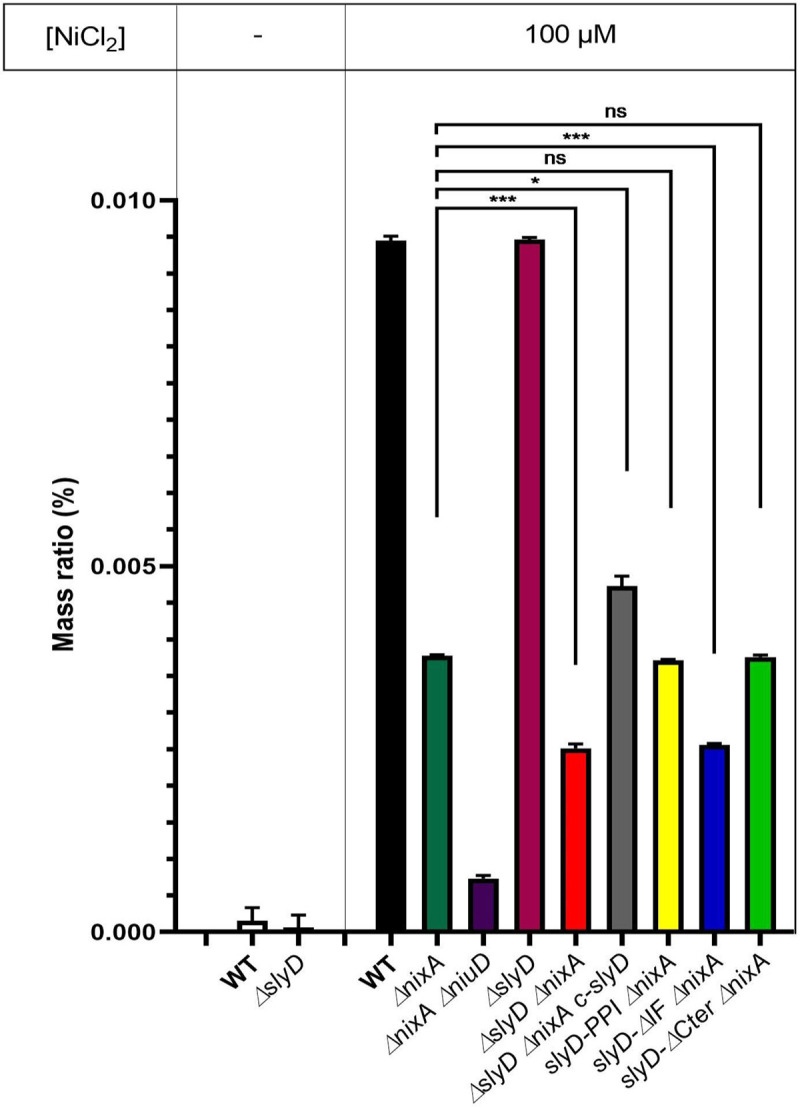
Measurement of the intracellular nickel content of *H*. *pylori* wild type strain and isogenic mutants by ICP-OES. Nickel amounts were measured by Inductively Coupled Plasma Optical Emission Spectrometry (ICP-OES) and are expressed as the percentage of the ratio of nickel mass versus total sample mass. Strains were grown either without added nickel or with 100 μM NiCl_2_. Color codes of the bars are as in Fig 4. The measurement of each strain under each condition was performed in triplicates in two experiments. Statistics are presented only for the comparison with the *ΔnixA* mutant. * corresponds to p<0.05, *** to p<0.001 and "ns" for non-significant. S9 Fig presents the complete statistical analysis of these data.

To evaluate the role of SlyD in nickel accumulation more precisely, we measured the total intracellular nickel content of our collection of mutants grown 24h in the presence of 100 μM NiCl_2_, by Inductively-coupled plasma optical emission spectrometry (ICP-OES) as previously reported [13] (Fig 6). Strains grown without supplemented nickel had metal levels below the detection limit. In agreement with our previous publication [13], the nickel content of the *ΔnixA* and *ΔnixA ΔniuD* mutants was reduced 2.4-fold and 15-fold, respectively as compared to the wild type strain. Consistent with the data presented above, the *ΔslyD* mutant accumulated nickel as efficiently as the wild type strain. However, when the *ΔslyD* mutation was combined with *ΔnixA*, we measured a significant 1.5-fold and 3.5-fold reduction in nickel content as compared with the *ΔnixA* single mutant and the wild type strain, respectively. This reduction was clearly restored in the complemented *ΔslyD ΔnixA c-slyD* strain. When compared to the full-length deletion of *slyD*, only the *slyD-IF* mutant was significantly deficient in nickel accumulation. These data confirm that SlyD is indeed involved in nickel accumulation in *H*. *pylori* and underline a major role of its chaperon function associated with the IF domain.

### SlyD is required for the activity of the nickel ABC transporter Niu

Increased tolerance to nickel toxicity associated with decreased nickel availability and accumulation could result from impaired metal import, a reduction in intracellular nickel storage capacity or enhanced export. Concerning nickel efflux, the only published system is Czn [19]. However, in our strain and under our conditions, we did not detect any increase in nickel sensitivity in mutant strains lacking this system alone (*ΔcznABC*) or in combination with the *ΔslyD* deletion (*ΔcznABC ΔslyD*). This excludes a role of the Czn efflux system in our nickel tolerance phenotypes.

**Fig 7:**
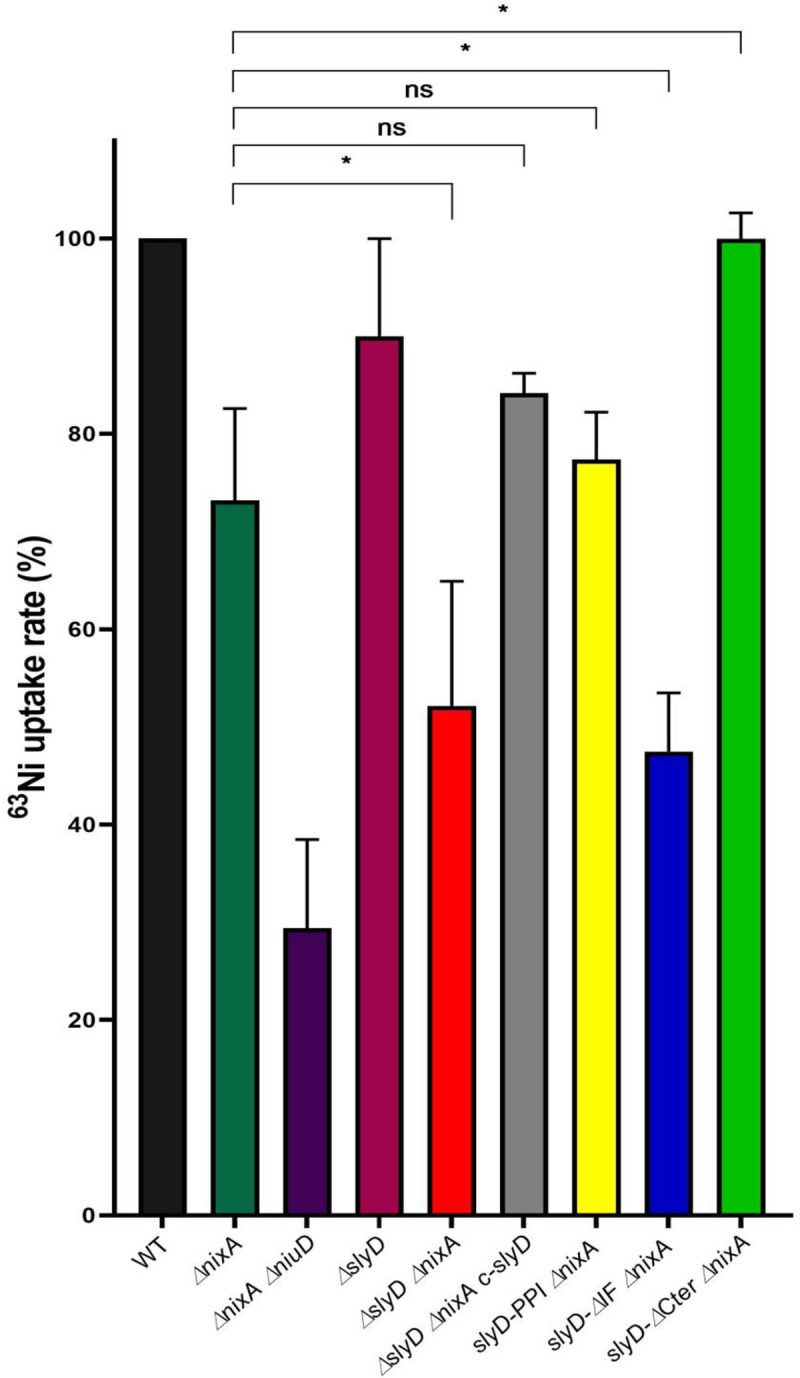
SlyD is required for the uptake of radioactive nickel by the Niu nickel transporter. Measurements of radioactive nickel uptake rates in *H*. *pylori* B128 wild type strain and isogenic mutant strains in the presence of 10 μM of ^63^NiCl_2_. Uptake rates are normalized to the rate of wild type *H*. *pylori* strain. Color codes of the bars are as in Fig 4. Error bars represent the standard deviation. Statistics are presented only for the comparison with the *ΔnixA* mutant. * corresponds to p<0.05 and "ns" for non-significant. S9 Fig presents the complete statistical analysis of these data.

To define the role of SlyD in nickel import, the uptake rates of radioactive ^63^NiCl_2_ were measured in the wild type and mutant strains at pH 5 during 30 min (Fig 7). The nickel uptake rate is moderately reduced in the *ΔnixA* mutant and strongly decreased in the *ΔnixA ΔniuD* mutant [13]. No significant change in the nickel uptake rate was seen in the *ΔslyD* mutant compared to that of the wild type bacteria. However, the *ΔslyD ΔnixA* mutant presented a 50% reduction in ^63^Ni uptake rate, which was significantly different from the rate of the *ΔnixA* mutant and could be complemented with wild-type *slyD*. The three strains carrying targeted *slyD* mutations in the *ΔnixA* background were also tested. The *slyD-ΔIF ΔnixA* strain exhibited a reduced uptake rate that phenocopied that of the *ΔslyD ΔnixA* mutant. While the *slyD-PPI ΔnixA* behaved like the *ΔnixA* mutant, we observed that nickel uptake of strain *slyD-ΔCter ΔnixA* was more robust than the *ΔnixA* mutant and closer to the level of the WT strain.

These results demonstrate that, under these test conditions, SlyD is required for optimal nickel uptake by the Niu transporter and only its chaperone domain plays a crucial role in this process.

### By which mechanism does SlyD impact the activity of the Niu transport system?

The finding that a PPIase like SlyD regulates a metal transport activity has, to our knowledge, never been previously reported. Therefore, several tests were performed in order to investigate how SlyD regulates the Niu transport system. First, we examined whether SlyD controls the expression of the *niu* genes. Expression of the *niuD* and *niuB1* genes (respectively, the first gene of the *niuDE* operon and the first gene of the *niuB1-niuB2* operon encoding the two NiuB paralogous proteins [13]), as monitored by RT-qPCR, were similar in the wild type and *ΔslyD* mutant (S4 Fig). This indicates that SlyD does not regulate the Niu transporter at the transcriptional level.

We then examined whether SlyD impacts the protein levels of components of the Niu system or their subcellular localization (S5 Fig). To this aim, the NiuB1 protein was purified and used to produce specific polyclonal antibodies. Contrary to NiuB1, attempts to produce anti-NiuD antibodies were unsuccessful. Therefore, a C-terminal fusion between the *niuD* gene and a V5-tag was constructed and introduced at its original locus into the wild type strain and the *ΔslyD* mutant. The amounts and localization of NiuD and NiuB were analyzed by Western blot after fractionation of crude extracts of bacteria grown without or with the addition of 100 μM NiCl_2_, a non-toxic nickel amount (S5 Fig). As previously reported, production of the NiuB and NiuD proteins was repressed by nickel [52,53]. Notably, SlyD impacted neither the amounts nor the localization of the two proteins. NiuB was found in both the soluble extract and inner membrane fractions in a NiuD-independent manner. As expected, NiuD was exclusively detected in the inner membrane.

These results show that SlyD impacts neither the protein levels nor the subcellular localization of NiuD and NiuB. This suggests that SlyD might regulate the activity of the Niu transporter by direct protein interaction.

### SlyD interacts with NiuD but not with NiuB or NiuE

We hypothesized that the control of the Niu transporter by SlyD might involve direct interaction between SlyD and one or more components of the Niu system. This possibility was examined by using the Bacterial Adenylate Cyclase Two-Hybrid system (BACTH) and testing pairwise interactions between protein fusions in *E*. *coli* [54]. The *slyD* gene was fused at its 5’ or 3’-extremity with a fragment encoding the T25 domain of the *Bordetella pertussis* adenylate cyclase into the pKT25 or pNKT25 vector, respectively. The *niuB1*, *niuE*, and *niuD* genes were fused at their 3’-extremities with the T18 fragment adenylate cyclase into the pUT18 vector. Fusions with the 5'-extremity of NiuD could not be obtained as they were toxic in *E*. *coli*.

**Fig 8:**
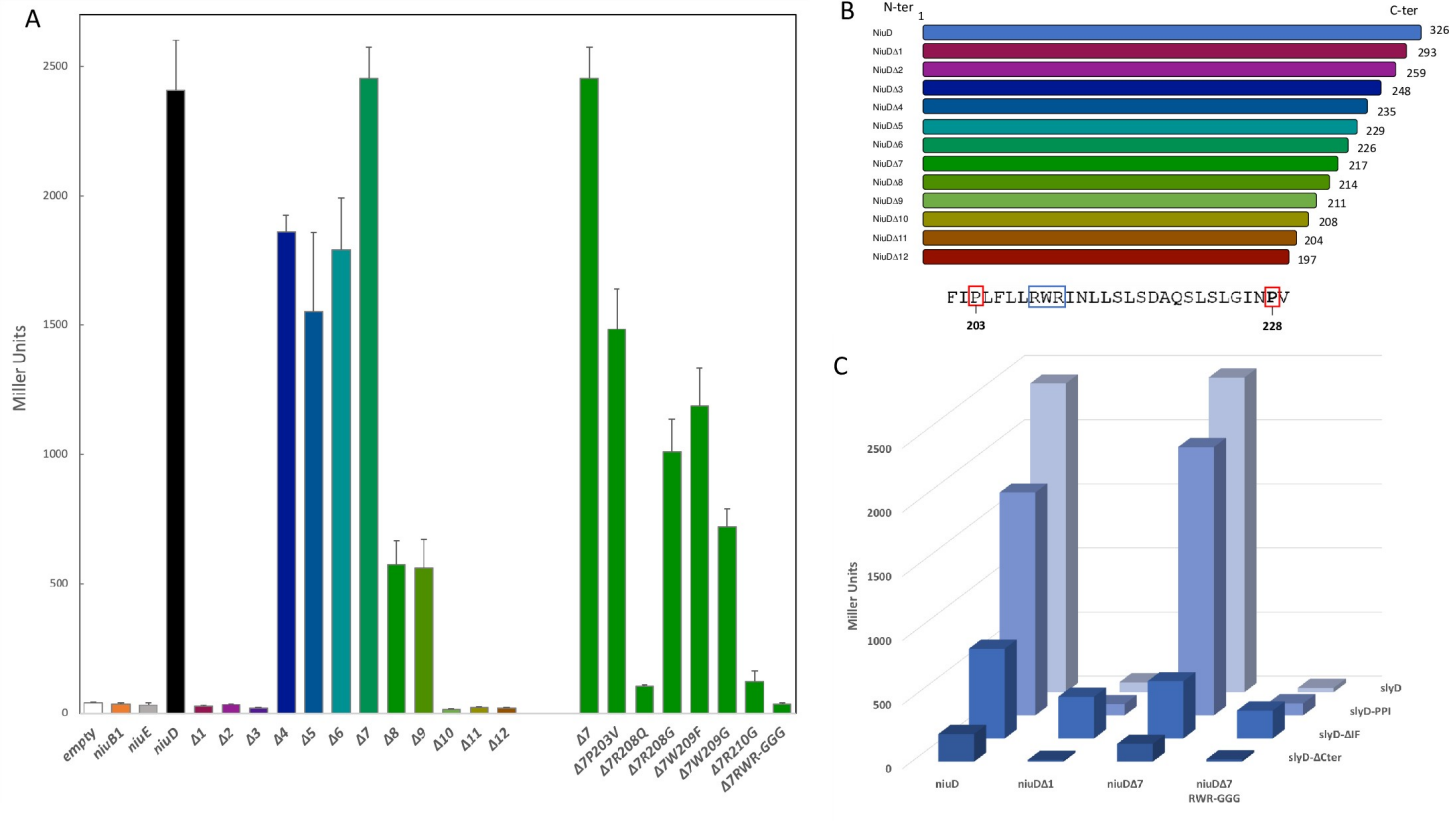
SlyD interacts with NiuD, the membrane permease of the Niu nickel uptake system. Bacterial two-hybrid BACTH was used to analyze, in *E*. *coli* strain BTH101, the interaction between SlyD and the NiuD permease. The values and standard deviation for each strain and controls are available in S6 Table, and each measurement was performed three times. **A.** ß-galactosidase activities of pairwise combinations of WT SlyD with different truncated and mutant versions of the NiuD protein. **B.** Illustration of the truncated NiuD protein versions and sequence of the NiuD region surrounding the RWR motif in the region that is required for the interaction with SlyD. **C.** ß-galactosidase activities of pairwise combinations of wild type and mutant SlyD proteins (PPI, ΔIF and ΔCter) with wild type and mutant NiuD proteins.

No interaction was detected between SlyD and either the periplasmic nickel-binding protein NiuB1 or the cytoplasmic NTP-binding protein NiuE. In contrast, combinations of pNKT(*slyD*) with pUT18(*niuD*) (in which the 3'-extremity of *niuD* is fused to the T18 sequence) scored positive in the ß-galactosidase assay revealing an interaction between SlyD and the transmembrane permease subunit NiuD (Fig 8A). As a control, we verified that NiuD was correctly targeted to the inner membrane in *E*. *coli* (S6 Fig). To delineate the region of interaction of NiuD with SlyD, we constructed plasmids expressing T18 fusions of NiuD proteins with progressive C-terminal truncations (Figs 8A and 8B and S7). The first NiuD deletions (Δ1, Δ2, Δ3) abolished its interaction with SlyD and larger deletions (Δ4, Δ5, Δ6, Δ7, Δ8, Δ9) restored the interaction with SlyD. These results can be interpreted in light of a predictive model of the NiuD protein folding and trans-membrane segments (S7 Fig). Indeed, this model suggests that the Δ1, Δ2, Δ3 fusions are predicted to expose the T18 fused domain into the periplasm, a localization that prevents its interaction with the cytoplasmic SlyD-T25 fusions. In contrast, the larger deletions (Δ4, Δ5, Δ6, Δ7, Δ8, Δ9) are compatible with a cytoplasmic exposure of the T18 domain. These interpretations also reinforce our confidence in the specificity of the NiuD-SlyD interaction. When we further deleted the NiuD protein (Δ10, Δ11, Δ12), the interaction was lost. The comparison between Δ9 (positive for interaction) and Δ10 (negative for interaction) allowed us to identify, at the NiuD C-terminus, a predicted cytoplasmic loop with three residues (Arg208-Trp209-Arg210) that are essential for NiuD interaction with SlyD.

To further determine which NiuD residues are important for its interaction with SlyD, we introduced mutations into plasmid pUT18(NiuDΔ7) that targeted the region defined above (Figs 8A and 8B and S7). First, when we introduced a triple exchange of Gly for residues Arg208-Trp209-Arg210, the ß-galactosidase activity dramatically dropped, confirming the importance of this region for NiuD interaction with SlyD. In the cases of single mutants Pro203 to Val, and Trp209 to Phe or Gly, the ß-galactosidase activity decreased but was still significant, indicating that these residues are not crucial for the NiuD interaction with SlyD. In contrast, the interaction was abolished when we introduced into NiuD the Arg208 to Gln and Arg210 to Gly mutations, indicating that these two arginine residues are essential for the NiuD interaction with SlyD.

Finally, we checked the impact of SlyD mutations on the interaction with full-length NiuD and Δ7, which generated comparable results, and used Δ1 and the triple RWR mutant as negative controls (Fig 8C). For the NiuD and Δ1 constructs, the interaction is maintained with the SlyD-PPI mutant and decreased with SlyD-IF. For the SlyD-Cter mutant, although the ß-galactosidase activity was diminished, the interaction was still detectable above baseline, and the reduced signal may be due to the reduced expression of this fusion given that the stability of this SlyD mutant protein is reduced (S6 Fig). Unexpectedly, the SlyD-IF mutant displayed significant interaction with the NiuDΔ1 and triple mutant negative controls suggesting that the IF chaperone region of SlyD contributes to the specificity to its protein interaction.

These data revealed a physical interaction between the SlyD protein and the NiuD permease subunit and defined regions of the transporter that are critical for this direct contact.

### SlyD is essential for the mouse stomach colonization

**Fig 9:**
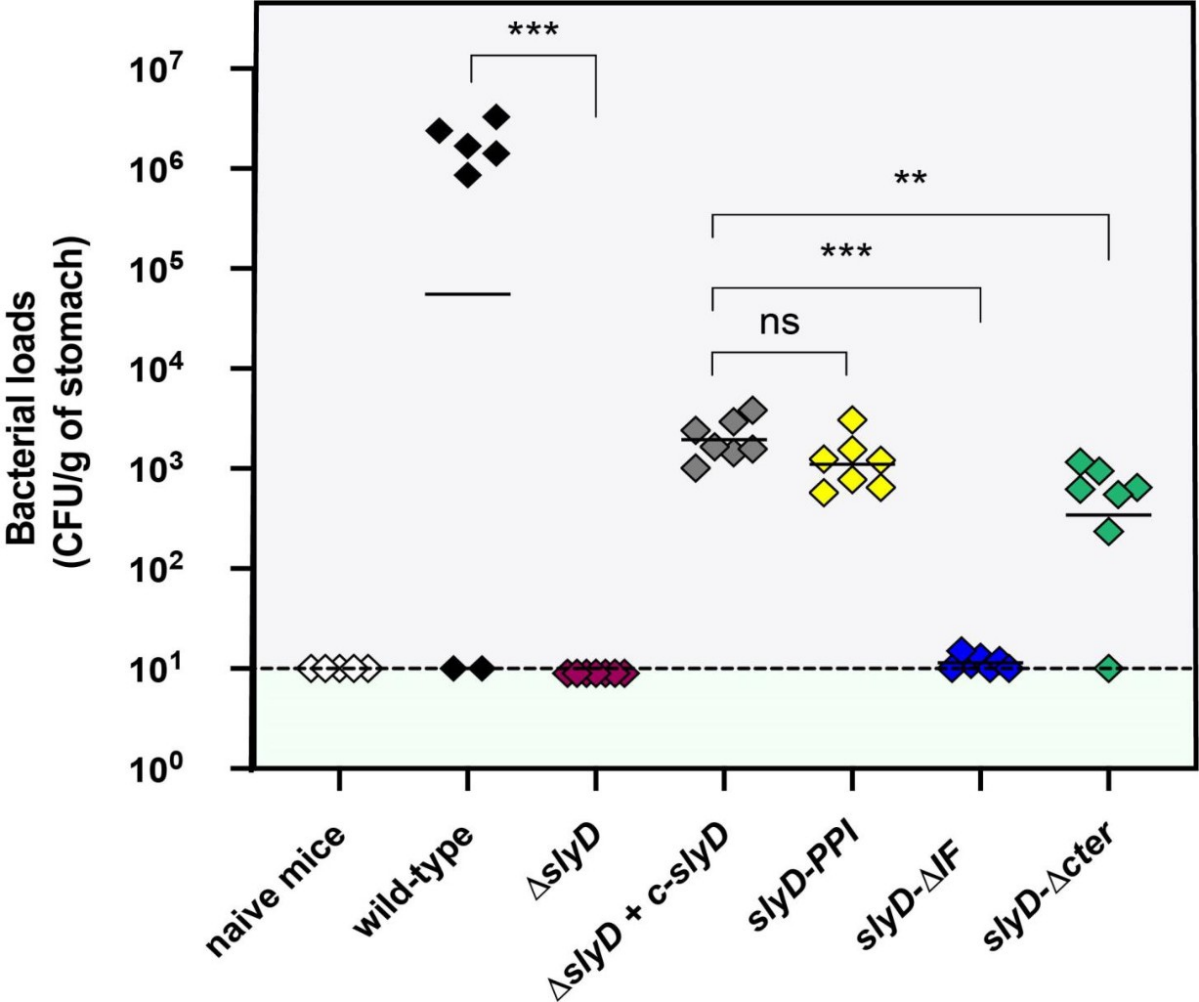
SlyD is essential for mouse colonization by *H*. *pylori* SS1 strain. Each diamond corresponds to the colonization load of one mouse one month after infection with the *H*. *pylori* strain indicated below. Each strain was tested in a group of seven mice. The color codes for the different strains are as in Fig 4. Horizontal bars represent the geometric means of the colonization load for the wild type bacteria, each mutant and the chromosomally complemented *ΔslyD* mutant (designated *c-slyD*). A dashed line shows the detection limit. The Δ*slyD* and isogenic mutants exhibited a statistically significant colonization defect when compared to the WT or *ΔslyD c-slyD* strains, respectively (**p<0.01, ***p<0.001 and "ns" non-significant).

The role of SlyD during gastric colonization was evaluated using the mouse model of infection by *H*. *pylori*. Different *slyD* mutations were introduced into the *H*. *pylori* mouse-adapted strain SS1 [55]; these included *ΔslyD*, the complemented strain *ΔslyD-c-slyD* and the *slyD-PPI*, *slyD-ΔIF* and *slyD-ΔCter* mutations. The expression of the SlyD protein in the WT and mutant strains was validated by western blot using anti-SlyD antibodies (S8 Fig). We orogastrically inoculated 10^9^ bacteria of WT SS1 and every mutant strain in seven NMRI mice each. One month later, colonization was assessed by quantitative cultures of stomach homogenates. As presented in Fig 9, the Δ*slyD* mutant was completely deficient in its capacity to colonize the mouse stomach. The complemented strain SS1 *ΔslyD+c-slyD* recovered partial capacity of colonization. This was also the case for the SS1 *slyD-PPI* and SS1 *slyD-ΔCter* mutants. In contrast *slyD-ΔIF* mutant was completely unable to colonize the mouse stomach just like the *ΔslyD* mutant.

These results show that *slyD* is indispensable for colonization of the mouse stomach. The PPI and C-terminus domains are not essential for *in vivo* colonization. In contrast, the chaperone domain plays a major role in gastric colonization.

## Discussion

Control of essential metal homeostasis is a complex and critical process. Bacteria have developed two major strategies to maintain this equilibrium. First, bacteria produce in the cytoplasm and/or the periplasm several "sequestering" proteins that specifically bind and store metals, preventing their association with unwanted targets [2]. The second strategy is control of metal import and/or efflux. In the vast majority of cases, expression of the metal transport systems is under the control of specific transcriptional regulators which have activities proportional to intracellular metal concentrations [2]. However, this type of regulation involves several steps as compared to post-translational regulation that was, to our knowledge, never reported for metal transporters in bacteria.

Here, such a novel mode of regulation was demonstrated; we found that the SlyD metallochaperone is required for the activity and thus active conformation of Niu, an essential nickel ABC transporter of *H*. *pylori*. In addition, SlyD is crucial for colonization of a mouse model by this pathogen. Our findings represent the first example of post-translational regulation of a metal transporter that relies on a peptidyl-prolyl isomerase (PPIase)-chaperone SlyD protein. To our knowledge, there is only one previous report of *cis-trans* Proline isomerization being required for activation of an ABC transporter [56]. In that instance, it was found that the periplasmic binding protein component of the system requires isomerization but the corresponding PPIase was not identified. Enzymes that combine chaperone and PPIase activities have mainly been characterized in *E*. *coli* for which nine such proteins were reported [25]. Their general function is to accelerate protein folding, but actual substrates are mostly unknown. One exception is the cytoplasmic Trigger factor (TF), a ribosome-associated molecular chaperone that, by acting as a foldase on numerous nascent polypeptides, protects them from misfolding and aggregation. TF mutants reported so far did not discriminate between the chaperone and PPIase activities and the PPIase activity is thought not to be central in bulk TF function [57]. In *H*. *pylori*, only three chaperones with PPIase activity are found, the periplasmic SurA homologue (HP0175) [58] and two cytoplasmic proteins: a TF homologue [59] and SlyD [24].

Here, we explored the *in vivo* role of *H*. *pylori* SlyD, a multifaceted protein that combines chaperone, PPIase and metal-binding properties. Using a collection of *H*. *pylori slyD* mutants, we characterized the function of this protein both *in vitro* and *in vivo*. We found that, *in vitro*, the SlyD PPIase activity was comparable to that of *E*. *coli* SlyD, and validated three essential active site residues. In our test, deletion of the IF chaperone domain did not affect PPIase activity. This result contrasts with previous studies that found that the two domains act synergistically, with the IF region being required for PPIase catalytic efficiency [24,60]. This discrepancy can be attributed to differences between the assays used. In these latter reports, protein refolding was measured, which reflects both chaperone and PPIase activities, while our test, using an isolated proline-containing tetrapeptide, specifically assays PPIase activity.

Using four different *in vivo* tests, we consistently demonstrated that the SlyD protein is important for the activity of the NiuBDE nickel ABC transporter. Using two-hybrid experiments, we found a direct interaction between the NiuD permease subunit and SlyD. Multicomponent ABC transporters generally require molecular chaperones for correct insertion within membranes in order to prevent aggregation or accumulation of toxic "dead-end" intermediates [61]. However, we observed no significant change of NiuD protein levels in the *H*. *pylori* inner membrane of the *ΔslyD* mutant, suggesting that SlyD does not affect synthesis and membrane insertion of NiuD but that it likely acts beyond this step. We found that the chaperone activity of SlyD, located in the IF domain, is required for NiuD activity, probably by helping the permease to acquire an active conformation. Indeed, in our tests, the SlyD-ΔIF mutant phenocopied the *ΔslyD* mutant with respect to nickel uptake; this is not due to a defect in PPIase activity, since deletion of the IF domain (SlyD-ΔIF) does not affect the PPIase activity *in vitro*. In two-hybrid tests, the interaction between the SlyD-ΔIF mutant and NiuD was reduced and the SlyD-ΔIF mutant presented, with negative controls, presumably non-specific interactions. We conclude that the SlyD IF domain is required for a productive NiuD-SlyD interaction and activation and, more generally, that the IF domain might be important for SlyD target recognition and discrimination. The function of the SlyD PPIase activity in the activation of Niu is more intriguing. Indeed, among the four tests that we performed, the *slyD-PPI* mutant only robustly phenocopied the *ΔslyD* mutation in the assay of tolerance to the high 1.5 mM nickel concentration. In the other tests, where nickel concentration was much lower (100 μM and 10 μM), the SlyD-PPI mutant did not display a major loss of Niu activation. In addition, as suggested by the results of the two-hybrid assays, the nickel tolerance phenotype of the SlyD-PPI mutant is not a consequence of a loss of physical interaction between SlyD-PPI and NiuD. Thus, we conclude that the PPIase function of SlyD only becomes critical *in vivo* under conditions of nickel overload, possibly through regulation of the NiuD conformation under these conditions (for instance through isomerization of the bonds of two proline residues close to the interacting region that we defined by two-hybrid) but it might also result from a role in the function of other nickel-resistance factors.

We also investigated the role of the C-terminal nickel-binding region of SlyD in Niu activation. This was particularly interesting since nickel binding at the SlyD C-terminal region was reported to inhibit PPIase activity of the *E*. *coli* protein [25] and Cheng *et al*. [24] observed conformational changes around the FKBP/PPIase domain after nickel binding to *H*. *pylori* SlyD. *In vitro*, we found that removal of the SlyD C-terminal region resulted in increased *in vitro* PPIase activity. Furthermore, addition of an equivalent of nickel completely inhibited the PPIase activity of the wild type SlyD protein and this inhibition was preserved in the SlyD mutant lacking a C-terminal region. This result indicates that down-regulation by nickel is not only relying on the C-terminal region but also on other binding sites in the SlyD N-terminal domains. In future experiments, it will be interesting to define which residue(s) of the N-terminal SlyD domains is(are) required for this C-terminal independent inhibition.

Analysis of the phenotype of the SlyD C-terminal deletion mutant in *H*. *pylori* reveals that this region of the protein is not required for the impact of SlyD on nickel metabolism. Thus, the diminished ß-galactosidase activity measured when SlyD-ΔCter was tested with NiuD in two-hybrid assays was unexpected. However, we could attribute this result to a lower level of expression of the SlyD-ΔCter mutant under these test conditions. Interestingly, the SlyD-ΔCter mutant presented slightly higher radioactive nickel uptake rates. We speculate that the C-terminal SlyD region could negatively regulate its chaperone activity, an aspect that we intend to analyze in future studies.

The two-hybrid assays allowed us to define regions of NiuD that are important for its interaction with SlyD (Figs 8 and S7). We defined a three amino-acid long motif of NiuD (RWR), predicted to be located within a cytoplasmic loop between transmembrane helices 6 and 7, that is essential for its interaction with SlyD. Interestingly, this loop (and this RWR motif) is surrounded by two proline residues (Pro203, Pro228) that might be substrates for the PPIase SlyD activity. We speculate that isomerization of the corresponding AA-proline bonds might induce conformational changes either in the cytoplasmic loop containing the RWR motif, or in the flanking transmembrane helices, which could positively impact NiuD activity.

Finally, we demonstrated that SlyD is essential for colonization of the mouse stomach by *H*. *pylori*. PPIases belonging to other families have been associated with the virulence of several bacteria [62,63]. In the mouse model, we found that the chaperone function of SlyD is essential and observed minor effects of the PPI and ΔCter mutations. We previously published that the Niu transporter is essential for colonization [13]. Thus, it is likely that the essentiality of SlyD *in vivo* is, at least partially, due to defective nickel uptake by the Niu system. However, it is possible that additional *in vivo* essential functions are disturbed in the absence of SlyD.

**Fig 10:**
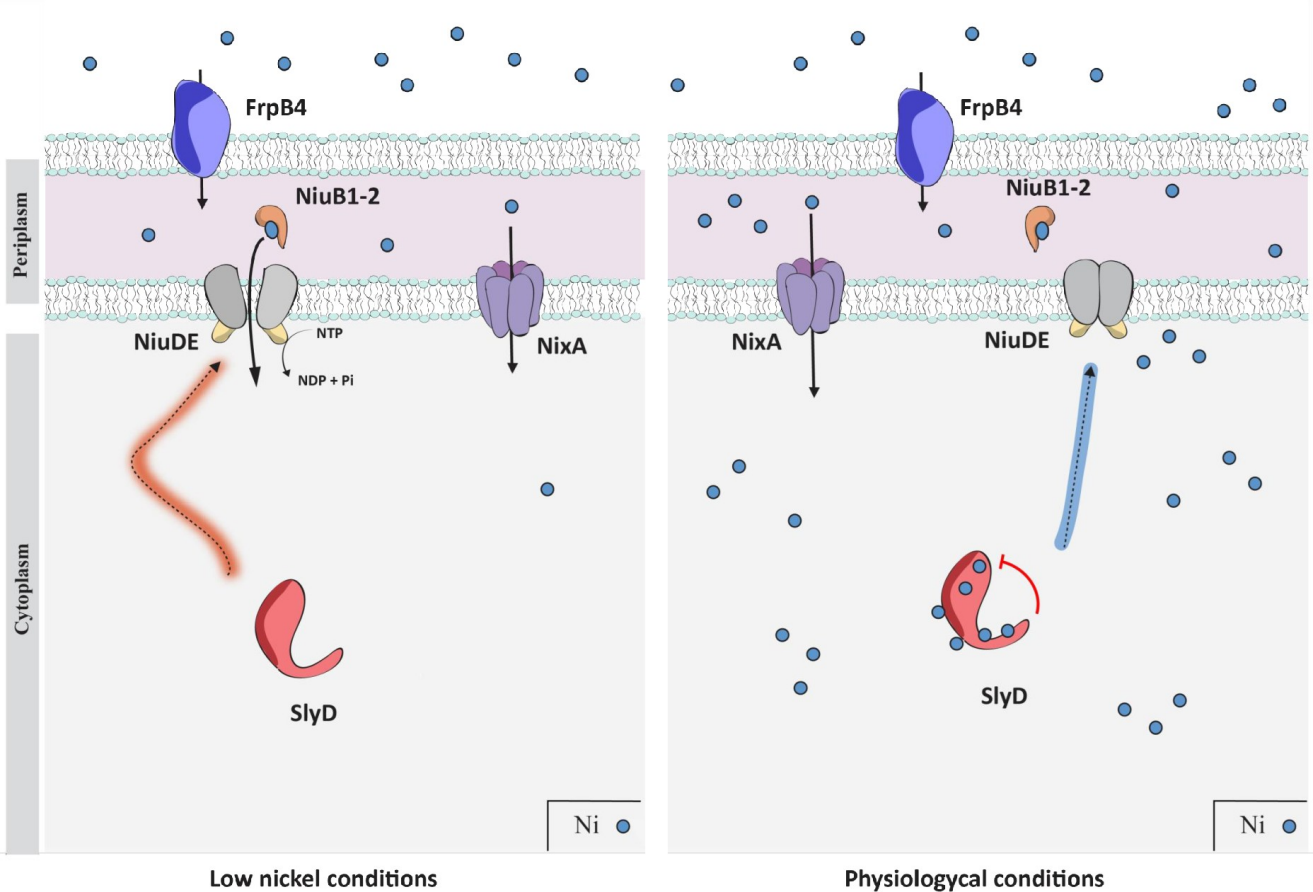
Model for the regulation of nickel uptake by the SlyD protein. In *H*. *pylori*, nickel ions (small blue dots) are transported across the outer membrane by FrpB4 (blue), a TonB-dependent transporter. Once in the periplasm, uptake of nickel through the inner membrane can be performed by the NixA permease (violet) or the ABC-transporter, NiuBDE (orange, grey and yellow). NiuB1-2 (orange) are the periplasmic nickel shuttles that deliver the metal to the NiuD permease, which activity is energized by the NiuE NTPase. The function of the Niu transporter requires activation by SlyD (red), a regulation that relies on direct interaction between SlyD and the NiuD permease. This is illustrated on the left panel, presenting conditions of low nickel availability. On the right panel, when nickel is available, binding of nickel to SlyD regulates its PPIase activity. This allows SlyD to sense the intracellular nickel concentration and to act as a gate keeper to control nickel entry.

We propose a model for the regulation of the NiuD nickel permease by SlyD that implies direct protein interaction (Fig 10). The SlyD chaperone activity is essential for the correct folding of NiuD or its association with its partners and thus for the functioning of the Niu transporter. SlyD could, in addition, play the role of a sensor of intracellular nickel concentration and act as a "gatekeeper" of nickel uptake allowing a shut down or activation of the Niu transporter when nickel bio-availability increases or decreases, respectively.

In conclusion, the role of SlyD in *H*. *pylori* is reminiscent of the multiple signaling roles of eukaryotic PPIases in cellular processes including immune response, neuronal differentiation and cell cycle control. We anticipate that bacterial PPIases have similar important properties that have, till now, been largely underestimated.

## Material and methods

### Ethics statement

Experiments in mice were carried out in strict accordance with the recommendations in the Specific Guide for the Care and the Use of Laboratory Animals of the Institut Pasteur, according to the European Directive (2010/63/UE) and the corresponding French law on animal experimentation (Arrêtés de 1988). The protocol has been approved by the Committee of Central Animal Facility Board of the Institut Pasteur (#08–551). To follow the new European directives, the project was approved by the CETEA, Comité d’éthique en Expérimentation Animale of the Institut Pasteur (#2013–0051) and by the Ministère de l’Enseignement Supérieur et de la recherche (#751501).

### Bacterial strains and growth conditions

The *H*. *pylori* strains used in this study are derivatives of B128 [47,48] and SS1 [55] (S2 Table). They were grown at 37°C under microaerophilic conditions (6% O_2_, 10% CO_2_, 84% N_2_) on Blood Agar Base 2 (Oxoid) plates supplemented with 10% defibrinated horse blood or Brucella broth agar (BD Difco) plates (designated BB) supplemented with 10% fetal calf serum (FCS, Eurobio). For liquid cultures, we used Brucella broth (BD Difco), designated BB, supplemented with 10% fetal calf serum (Eurobio) or with 0.2% β-cyclodextrin (Sigma), designated BBß. All plates and liquid cultures were supplemented with the following antibiotics-anti-fungal cocktail: amphotericin B 2.5 μg.mL^-1^, polymyxin B 0.31 μg.mL^-1^, trimethoprim 6.25 μg.mL^-1^ and vancomycin 12.5 μg.mL^-1^. Selection of *H*. *pylori* mutants and transformants was performed using kanamycin 20 μg.mL^-1^, chloramphenicol 6 μg.mL^-1^, streptomycin 10 μg.mL^-1^ or apramycin 10 μg.mL^-1^. *Escherichia coli* XL1-Blue bacteria grown on solid or liquid Luria-Bertani (LB) medium was used for subcloning and as a host for the preparation of the plasmids employed to transform *H*. *pylori*. *E*. *coli* strain BTH101 was used for Bacterial Two Hybrid and BL21(DE3) *ΔslyD*::*apra* for protein overexpression and purification (S2 Table). LB medium was supplemented with chloramphenicol 30 μg.mL^-1^, ampicillin 100 μg.mL^-1^ or kanamycin 40 μg.mL^-1^ when required.

### Molecular technics

Molecular biology experiments were performed according to standard procedures and the supplier (Fermentas) recommendations. NucleoBond Xtra Midi Kit (Macherey-Nagel) and QIAamp DNA Mini Kit (Qiagen) were used for plasmid preparations and *H*. *pylori* genomic DNA extractions, respectively. PCR was performed either with DreamTaq DNA polymerase (ThermoFisher), Q5 DNA polymerase (Biolabs) or with PrimeSTAR Max DNA polymerase (Takara) when the product required high fidelity polymerase. The pGEMT vector (Promega, S3 Table) was used to construct, in *E*. *coli*, the suicide plasmids that served for mutagenesis in *H*. *pylori*.

### Construction of *H*. *pylori slyD* mutants and of the *niuD-V5* fusion strain

An unmarked *slyD* deletion mutant of *H*. *pylori* strain B128 (S2 Table) was constructed by allelic exchange as previously described [13]. We used a *H*. *pylori* suicide plasmid derived from pGEMT, in which about 500 bp of the 5’-end and the 3’-end regions immediately flanking the open reading frame of *slyD* gene were cloned on each side of a *difH-cat-rpsL-difH* cassette amplified with primers difH-rpsL-cat1 and difH-rpsL-cat2 (primers are listed in S4 Table). This plasmid (S3 Table) was used to naturally transform *H*. *pylori* strain B128 that we made Streptomycin resistant. The insertion of the cassette by homologous recombination was selected on blood agar plates containing chloramphenicol 6 μg.mL^-1^. Removal of the cassette was achieved by plating the Cm^R^ clones on blood agar plates containing streptomycin 10 μg.mL^-1^. The Gibson assembly method was used to obtain a PCR product designed to introduce a *slyD* deletion in *H*. *pylori* strain SS1 (S2 Table). This PCR product carried a Cm resistance cassette flanked by 500 bp upstream and downstream *slyD* PCR fragments. Deletion of the *slyD* gene was verified in both genetic backgrounds by PCR and sequencing of the gene region. Plasmids used for these constructs are listed in S3 Table. The *slyD* deletions of strains B128 and SS1 were complemented by reintroducing a wild type *slyD* copy at its original locus on the chromosome under the control of its own promotor. For that, a PCR product was generated with the Gibson assembly procedure. This PCR product comprised the *slyD* gene followed by the apramycin resistance cassette and flanked by the 500 bp upstream and downstream flanking region of the *slyD* gene. The final PCR-amplified product was used to directly transform in B128 *ΔslyD* or SS1 *ΔslyD*::*Km* strain resulting in a strain in which the wild type *slyD* gene and the *apra* cassette integrated by homologous recombination between flanking regions of the *slyD* locus. The same procedure was used to introduce *slyD* versions carrying mutations (PPI, ΔIF, ΔCter) in strain B128 and SS1. Sequencing was performed in both genetic backgrounds to verify the deletions and reintroduction of wild type or mutated versions of *slyD* at the correct locus. The expression of the mutated SlyD versions in B128 and SS1 was validated by western blot.

The NiuD-V5 fusion was obtained by gene synthesis (Eurofins). A PCR fragment carrying this fusion, a kanamycin resistance cassette and 500 bp downstream the *niuD* gene were fused in this order using the Gibson assembly procedure. The final PCR product was directly naturally transformed into *H*. *pylori* and allelic exchange was selected on kanamycin. Correct insertion of the fusion and cassette were verified by PCR and sequencing.

### Purification of recombinant *H*. *pylori* SlyD proteins and circular dichroism spectroscopy analysis

SlyD wild-type (WT) and mutants (PPI, ΔIF and ΔCter) proteins were expressed according to the method previously described [38] in a Δ*slyD*::*apra* BL21(DE3) strain of *E*. *coli* using pET28(a)+ vector (Novagen). SlyD proteins were purified by using a nickel-nitrilotriacetic acid (Ni-NTA, Qiagen) column and the presence of SlyD in the protein fractions collected at each step was verified by SDS-PAGE analysis. Cleavage with thrombin was initiated by the addition of 0.3 U thrombin protease (Novagen) per mg target protein. The purified proteins were dialyzed overnight in thrombin cleavage buffer (20 mM Tris-HCl, pH 8.4, 150 mM NaCl, 2.5 mM CaCl_2_) for 12 hours at 4°C. This step was followed by anion exchange on a MonoQ column (GE Healthcare) in Tris-HCl 20mM + 200mM NaCl, 1mM TCEP buffer. Selected fractions were pooled and used for verification by Mass Spectrometry analysis (Department of Chemistry, University of Toronto) and further activity assays.

CD spectra were recorded on an Olis rapid scanning monochromator at room temperature. Approximately 15 or 25 μM protein was used in 5 mM Phosphate buffer, pH 7.6. CD spectra were collected by scanning the wavelength range of 200–260 nm using a step size of 1 nm and an integration time of 2 s. Five scans were averaged for each sample, and each CD spectrum was normalized to mean residue ellipticity [θ]_mre_ (deg cm^2^ dmol^–1^) using the equation [θ]_mre_ = [(MM/*N*– 1) × θ]/(*c* × *l* × 10)], where MM is the molecular mass of the protein in Da, *N* is the number of residues, θ is the measured ellipticity (degrees), *c* is the total protein concentration in g/mL, and *l* is the cell path length. The averaged spectra were smoothed by using a three-period moving average. The concentrations of the SlyD samples were confirmed after the scans.

### Peptidyl-prolyl isomerase activity assays

An uncoupled protease-free assay was used to measure the PPIase activity of SlyD and the variants [40,50]. The substrate, succinyl-Ala-Phe-Pro-Phe-4-pNa (Bachem Bioscience), was dissolved in trifluoroethanol (dried over sieves) and 0.47 M LiCl (dried in 220°C oven overnight). The reactions contained 35 mM HEPES, pH 7.6, and 2 μM of each purified SlyD protein (wild type or mutants) and were incubated at 10°C before the addition of 71 μM substrate with/out 2 or 100μM of NiSO_4_. Isomerization was monitored at 314 nm at 10°C on a Cintra 404 spectrophotometer and fit to a single exponential decay. The time course of the reversible first-order prolyl isomerization was measured during 180 seconds, and the apparent second-order rate constant was calculated.

### Metal sensitivity assay and evaluation of intracellular nickel content

The effect of metal exposure on *H*. *pylori* growth was tested by inoculating bacteria at OD_600_ 0.1, in 10 mL liquid medium (BB with FCS) without or with 1.5 mM NiCl_2_. Bacterial growth was monitored 24 hours later by measuring their OD_600_. The data correspond to at least three independent experiments.

For the evaluation of the intracellular nickel content, we used a P_*fecA3*_ promoter fusion as a reporter, as previously validated [13]. The P_*fecA3*_ promoter is under the control of the nickel-responsive transcriptional regulator of *H*. *pylori*, NikR and its activity is thus proportional to the intracellular nickel concentration. A pILL2157(P_*fecA3*_::*lacZ)* fusion plasmid (S3 Table) was transformed into strain B128 and its isogenic mutants. To measure the activity of the reporter gene, *H*. *pylori* bacteria were grown on blood agar plates for 24 hours, then inoculated at OD_600_ 0.05 in BB FCS liquid medium and grown overnight. This preculture was used to inoculate the bacteria at OD_600_ 0.1 in liquid BB FCS without or with the addition of 100 μM NiCl_2_. After 24h, the β-galactosidase activity of these cultures was measured to monitor the response to nickel of the reporter fusion, and thus NikR activity (the values are available in S5 Table).

### Nickel content measurements by Inductively Coupled Plasma Optical Emission Spectrometry (ICP-OES)

Overnight liquid cultures of *H*. *pylori* strain were diluted and grown until OD_600_ 0.1 at 37°C in 15 ml Brucella-Broth with FCS, then 100 μM NiCl_2_ were added and the cultures were left to grow until OD_600_ 6 after 24h. Then, the cultures were washed once with PBS-1X prior to be resuspended in cold PBS-1X with EDTA 1mM and adjusted at OD_600_ 10. Six mL of this culture preparation were centrifuged at 4,000 g at 4°C for 25 min through 400 μL of a 1:2 mixture of the silicone oils AR20/AR200 (Wacker) in order to separate the cells from the medium. Pellet were dried by speed-vac for 2h at 60°C. Ten mg of the pellet were mineralized overnight with a solution mix of 500μL nitric acid 69% (EMSURE) and 500 μL sulfuric acid 96% (Alfa Aesar). After mineralization, MiliQ water was added in each sample to a final volume of 20 mL. Nickel content was measured by ICP-OES with an Agilent 720 Series with axially-viewed plasma and with a Ni calibration curve of 10–1,000 ppb at “Institut Lavoisier de Versailles”. The content of Ni(II) was determined using a curve established with certified ICP grade nickel-standards. The measurement of each strain under each condition was performed in triplicates in two experiments. The results are presented as the percentage of the ratio of nickel mass versus total sample mass.

### Transport of radioactive nickel

The procedure was adapted from our previously published protocol [13]. The preculture of B128 wild type and isogenic mutants was used to inoculate, at OD_600_ 0.1, 10 mL of fresh BB medium supplemented with 10% FCS and incubated under microaerophilic conditions with shaking at 37°C. When the cultures reached OD_600_ 0.5, cells were harvested, washed and resuspended in the same volume of BBβ and shaken during 20 min under microaerophilic conditions at 37°C. Radioactive ^63^NiCl_2_ (3.953 mCi/mL), was isotopically diluted 10-fold with cold NiCl_2_ and added at a final concentration of 10 μM. ^63^NiCl_2_ was supplied by the Eckert & Ziegler Isotope Products (Valencia, CA USA). Kinetics were performed for 30 minutes. Aliquots of 1 mL were withdrawn, immediately vacuum filtered through 0.45 μm pore-size cm filters (diameter = 2.5; Millipore) and washed with 10 mL of 50 mM HEPES buffer (pH 7.0), 1 mM cold NiCl_2_. Two series of experiments were performed and each time point was measured in duplicates. Uptake rates were calculated as CPM of accumulated ^63^Ni as a function of time.

### Urease and [NiFe] hydrogenase activity measurements in *H*. *pylori* strains

Urease activity of whole *H*. *pylori* cells was assayed by measuring the ammonia production using the Ammonia-Assay kit (Sigma) as described [13]. The NH_3_ concentration in the supernatant was measured with the ammonia-assay kit according to the manufacturer’s (Sigma) instructions. Hydrogen uptake activity was determined spectrophotometrically at 604 nm by following the color change of methyl viologen (MV) from a colorless oxidized form to a dark-violet reduced form as described in [20]. The data correspond to at least three independent experiments with two technical replicates each time.

### Bacterial Two-Hybrid assays

The Bacterial Two-Hybrid (BACTH) test is based on the reconstitution of adenylate cyclase activity in a *cya*^*-*^ *E*. *coli* strain as a result of the interaction between two proteins: a bait and a prey fused to two separate catalytic domains (T18 and T25) of the *Bordetella pertussis* adenylate cyclase. Empty pNKT25 an pUT18 vectors served as controls of background adenylate cyclase activity [54]. To detect interactions between the SlyD and Niu proteins, *slyD* and *niu* genes were amplified by PCR using primers listed in S4 Table and chromosomal DNA from B128 *H*. *pylori* strain as a template.

Several plasmids were constructed (S3 Table) expressing either an N-terminal or a C-terminal fusion of these proteins with the T25 catalytic domain (derived from vectors pKNT25 and pKT25, respectively) or either a N-terminal or a C-terminal fusion with the T18 catalytic domain (derived from vectors pUT18 and pUT18C, respectively). All inserts were digested by *XhoI* and *EcoRI*, and were then cloned into plasmids pUT18, pNKT25, pUT18C and pKT25 (PCR primers listed in S4 Table). The two plasmids expressing fusions to be tested were co-transformed in *E*. *coli* strain BTH101 and transformants were selected in Luria-Bertani agar plates containing kanamycin and ampicillin at 30°C. To avoid toxic effect of transformation of the plasmid pUT18(*niuD*^*+*^) into the recipient strain BTH101, 1% glucose was added to the media. Five mL of LB medium supplemented with antibiotics and IPTG 10^−3^ M were inoculated with the transformants clones and incubated overnight at 30°C. Quantification of the interactions in strains carrying each plasmid combination was obtained by measurement of the β-galactosidase activity expressed in Miller units that was performed in at least 5 replicates as in [54] (S6 Table).

### RNA extraction and cDNA synthesis

A total of 30 ml of three independent cultures grown at pH7 for 24 h with/out 100 μM NiCl_2_ was centrifuged for 15 min at 4000 *g*, treated with RNA protect solution (Qiagen) and stored at -80°C. Cells were lysed and RNA was extracted with the Nucleospin miRNA kit (Macherey-Nagel). RNA was incubated for 30 minutes at 37°C with 2 U/μL of Turbo DNase-free enzyme (Ambion). Synthesis of cDNA reactions were carried out following the manufacturer’s protocol using SuperScript IV First-Strand Synthesis System (ThermoFisher), starting with 1 μg total RNA. cDNA was final diluted to 10 ng/μl in Nuclease-free water. qRT-PCR Mix was performed with Power SYBR Green PCR Master Mix (Applied Biosystems), 900 nM of each primer (S4 Table), and 30 ng of total cDNA. PCR products were amplified and detected with an Applied Biosystem (Thermofisher) machine. The cycling conditions were as follows: one cycle at 95°C for 10min, 45 cycles at 95°C for 15 s and 60°C for 2 min, and 80 cycles at 55°C for 30 s with a 0.5°C increase every 30 s. The transcript levels were normalized to the level of the housekeeping *ppK* (encoding polyphosphate kinase, HP1010) as previously validated [18]. The data correspond at least two independent experiments with two technical replicates each time.

### Fractionation and western blots

Recombinant *H*. *pylori* SlyD protein was purified from *E*. *coli* and used to raise polyclonal antibodies that were validated by Western blot under reducing conditions (with DTT) (Fig 2A). The cellular fractionation protocol was adapted from [64]. *H*. *pylori* cells were grown to an OD_600_ of 0.8–1, then harvested by centrifugation and washed twice in PBS prior to be resuspended at OD_600_ 10 and disrupted by sonication in a lysis buffer containing 10 mM Tris-HCl pH7.5 (buffer A) and Complete Protease Inhibitor Cocktail (Roche). Cell debris was removed by centrifugation at 20,000g at 4°C for 15 minutes and supernatants were collected as total extracts. The supernatants were transferred to ultracentrifugation tubes (Polyallomer, Beckman Coulter) of 1.5 mL and then centrifuged 45 min at 100,000g at 4°C. The supernatant contains soluble fraction and the pellet total membranes. The pellet was washed once with buffer A and then resuspended in 10 mM tris-HCl pH 7.5 + 1% N-lauroyl-sarcosin (Sigma-Aldrich) and Complete Protease Inhibitor Complete (buffer B). After another ultracentrifugation step, the supernatant contains the inner membrane and pellet outer membrane.

Western blots were performed with 20 μg of proteins loaded and separated on a 4–20% Mini-Protean TGX Stain-Free precast protein gel (BioRad) and subsequently electrotransferred on a polyvinylidene difluoride (PVDF) membrane (Biorad) by TransBlot Turbo system (Biorad). The *H*. *pylori* SlyD and NiuB proteins were detected with rabbit polyclonal antibodies anti-SlyD and anti-NiuB at the respective dilutions of 1:5,000, 1:3,000. Goat anti-rabbit IgG-HRP (Promega) were used as secondary antibodies at 1:5,000 dilution and the detection were achieved with the ECL reagent (Thermo Fisher). V5 tag was detected with an antibody anti-V5 coupled with HRP (SantaCruz) at 1:10,000 dilution. The separation of the soluble and the membrane fractions was validated with a control Western Blot with antibodies against the cytoplasmic AmiE protein [65].

### Mouse model of colonization

NMRI-specific pathogen-free mice (Charles River Laboratories) were orogastrically inoculated with 10^9^ CFU of SS1 *H*. *pylori* WT and mutants prepared in 100 μL of peptone broth. One month after inoculation, mice were sacrificed and stomachs were harvested and crushed in peptone broth. Viable *H*. *pylori* colonizing the stomach were enumerated by the culture of serial dilutions of homogenized tissue on blood agar plates containing in addition bacitracin (200 μg.mL^-1^) and nalidixic acid (10 μg.mL^-1^).

### Statistical analysis

The Student's t-test was used to determine the significance of the means of the data. The Mann-Whitney test was used for mouse colonization assay to compare colonization loads.

## Supporting information

S1 Fig Multiple alignment of SlyD from different *H*. *pylori* strains and structural models of the *H*. *pylori* SlyD WT and mutant proteins. **A.** The alignment of the aminoacid sequence of a selection of SlyD proteins from 10 different *H*. *pylori* strains highlights a strong sequence conservation. Protein sequences were extracted from Kegg database and multiple alignment was made with Clustal Omega (https://www.ebi.ac.uk/Tools/msa/clustalo/). **B.** Prediction of the tridimensional structures of *H*. *pylori* mutant SlyD proteins. The NMR structure of *E*. *coli* SlyDΔCter protein (2KFW PDB [49]) served to represent its structure. When relevant, the C-terminal metal-binding region of unknown structure has been added for clarity (in green). The NMR structure of *H*. *pylori* SlyDΔCter protein [2KFW PDB [24]] served to represent its structure. Structure models of SlyD mutants was obtained with the Phyre2 software and modelized using PyMol. The PPIase domain is represented in blue, chaperone IF domain in pink and C-terminal region in green.
(PPTX)

S2 Fig Growth and viability of *H*. *pylori slyD* WT and mutant strains and subcellular localization of the SlyD protein. **A.** Growth curve and colony forming units (CFU) counts of WT and *slyD* mutant strains grown in BB medium at 37°C and followed during 1,500 min. Growth was followed by measuring OD_600_ and viability by CFU counting. The graphics represent a mean of three independent experiments and show that *slyD* deletion or mutations does not significantly impact *H*. *pylori* growth. **B.** Analysis of the subcellular localization of the SlyD protein in *H*. *pylori* by fractionation. Western blot was probed with anti-SlyD polyclonal antibodies on total extracts (T), soluble extract (S) and inner membrane (M) fractions prepared from *H*. *pylori* B128 wild type strain, a *ΔslyD* mutant and a *ΔslyD-c slyD* complemented strain. SlyD is exclusively detected in the soluble fraction, testifying of its cytosolic localization.
(PPTX)

S3 Fig Circular Dichroism analysis, PPIase activity and nickel regulation of *H*. *pylori* WT and mutant SlyD proteins. **A.** Circular Dichroism spectra of SlyD wild type and mutant proteins were analyzed in 5 mM phosphate buffer (pH = 7.6). Five scans were averaged for each sample. **B and C.** A protease-free assay was used to measure PPIase activity of purified WT and mutant SlyD proteins without nickel or after addition of NiSO_4_ at 2 μM (panel **B**) or 100 μM (panel **C**). PPIase activity of purified *E*. *coli* SlyD was measured as a control. The time course of the reversible *cis* to *trans* first-order prolyl isomerization of a tetrapeptide substrate was followed by the decrease of absorbance recorded at 314 nm and represented as ln(A/A_0_). The average of three independent experiments is represented.
(PPTX)

S4 Fig SlyD does not modify the expression levels of the *niuD* and *niuB1* genes in *H*. *pylori*. qRT-PCR normalized fold changes of the expression of *niuD* and *niuB1* genes in a B128 wild type strain and *ΔslyD* mutant. *ppK* was used as the housekeeping gene for normalization of the values. These results are the means with the standard deviations of two independent experiments.
(PPTX)

S5 Fig SlyD impacts neither the protein amounts nor the localization of NiuD and NiuB. Western blot analysis of total extracts (T), soluble extract (S) and inner membrane (M) fractions prepared from *H*. *pylori* B128 wild type strain and *ΔslyD* mutant, each expressing NiuD-V5 fusion, grown without or with 100 μM of NiCl_2_. Western blots were revealed with anti-NiuB (panel **A**) and anti-V5 antibodies (panel **B**). The amount of both NiuD and NiuB1 proteins was diminished in the presence of nickel as expected. NiuB protein (35 kDa) was found in both the soluble (that comprises the periplasmic fraction) and inner membrane fractions in similar amounts in the WT, Δ*slyD* and *ΔniuD* strains. NiuD-V5 protein (35 kDa) is exclusively localized in the inner membrane in similar amounts of WT strain and Δ*slyD* mutant.
(PPTX)

S6 Fig Controls for the BACTH assays: localization of the NiuD protein in *E*. *coli* and analysis of the production of SlyD WT and mutant fusions to the T25 fragment A. Western blot analysis of total extracts (T), soluble extract (SE) and inner membrane (IM) fractions prepared from *E*. *coli* BTH101 co-transformed with pU18::*niuDΔ5* and pNKT25::*slyD* or pNKT25::*slyD-PPI*. Western blot was revealed with anti-Cya antibodies. NiuDΔ5 fused to the T18 Cya fragment is located in the *E*. *coli* inner membrane. **B.** Western blot analysis of total extracts prepared from *E*. *coli* BTH101 carrying SlyD wild type and mutant proteins fused to the T25 fragment and revealed with anti-SlyD antibodies. The production of SlyD-ΔCter-T25 fusion is sharply diminished in comparison with WT SlyD-T25 fusion protein.
(PPTX)

S7 Fig Structural and topological features of NiuD and NiuD mutants used in the two-hybrid assay. **A.** Schematic representation of the predicated topology of a NiuD monomer as determined by the TMHMM program (*Upper panel*). N denotes the N-terminus, C the C-terminus of the protein; cylinders represent the 10 transmembrane helices (TMH) of NiuD and L1-L9 the periplasmic (L1, 3, 5, 7 and 9) and cytoplasmic (L2, 4, 6 and 8) loops (L) between TMHs. First and last residues flanking TMHs are indicated (numbering). L6’s sequence is indicated with the two proline residues in red and the RWR motif highlighted in blue; the RWR residues are depicted by blue circles on the scheme. *Lower panel* shows the structure prediction of a NiuD monomer (Phyre2 program, cartoon representation). TMH6 and 7 are green and L6 is orange, with the RWR motif (residues represented with blue sticks), and the two proline residues (spheres) flanking the loop in red. **B.** Schematic representation of NiuD’s and NiuD’s mutants (Δ1 to Δ12) topology. Each mutant’s name is indicated on the left and numbers indicate TMHs. The yellow circle depicts the T18 two-hybrid tag. In some cases, this tag might adopt alternative orientations/topologies that are indicated with grey cartoons. The periplasm is located above each figure, while the cytoplasm is located below. When T18 lies in or orientates towards the periplasmic side, it cannot participate to the interaction with SlyD-T25 that is located in the cytoplasm. Results of two-hybrid assays (SlyD-T25 and NiuD-T18 interactions) are indicated in each case with a color code.
(PPTX)

S8 Fig Western blot of equal amounts of total extracts, under reducing conditions, from the strains used for the mouse colonization. *H*. *pylori* SS1 WT strain and SS1-derived mutants carrying the following mutations *ΔslyD*, *ΔslyD c-slyD* (complemented strain), *slyD-PPI*, *slyD-ΔIF*, and *slyD-ΔCter* strain, that were probed with specific anti-SlyD polyclonal antibodies prepared during this study.
(PPTX)

S9 Fig Statistical analysis for the data of Figs 4, 5, 6 and 7. The Student's t-test was used to determine significant differences between every pair of mean values from Figs 4, 5, 6 and 7. A color code indicates the *p* values for each comparison. **A.** Statistical analysis of the values of tolerance to toxic nickel exposure of *H*. *pylori* wild type and mutants from Fig 4. **B.** Statistical analysis of the values of the nickel content reporter of *H*. *pylori* wild type and mutants from Fig 5. **C.** Statistical analysis of the values of the ICP-OES nickel content measurements of *H*. *pylori* wild type and mutants from Fig 6. **D.** Statistical analysis of the values of radioactive nickel uptake rates of *H*. *pylori* wild type and mutants from Fig 7.
(PPTX)

S1 Table Urease and hydrogenase activities of the WT B128 strain and its isogenic *ΔslyD* mutant. (DOCX)

S2 Table Strains used in this study. (DOCX)

S3 Table Plasmids used in this study. (DOCX)

S4 Table Oligonucleotides used in this study. (DOCX)

S5 Table ß-galactosidase activity, expressed in Miller units, by the P*fecA*::*lacZ* fusion in *H*. *pylori* wild type and mutants after 24 hours. (DOCX)

S6 Table ß-galactosidase activity, expressed in Miller units, of the two-hybrid assays. (DOCX)
