# Supplementary figures and images for: A novel mode of control of nickel uptake by a multifunctional metallochaperone
Source: PLoS Pathog. 2021 Jan 14;17(1):e1009193. doi: 10.1371/journal.ppat.1009193 (PMC7840056; doi:10.1371/journal.ppat.1009193)

## Slide 1
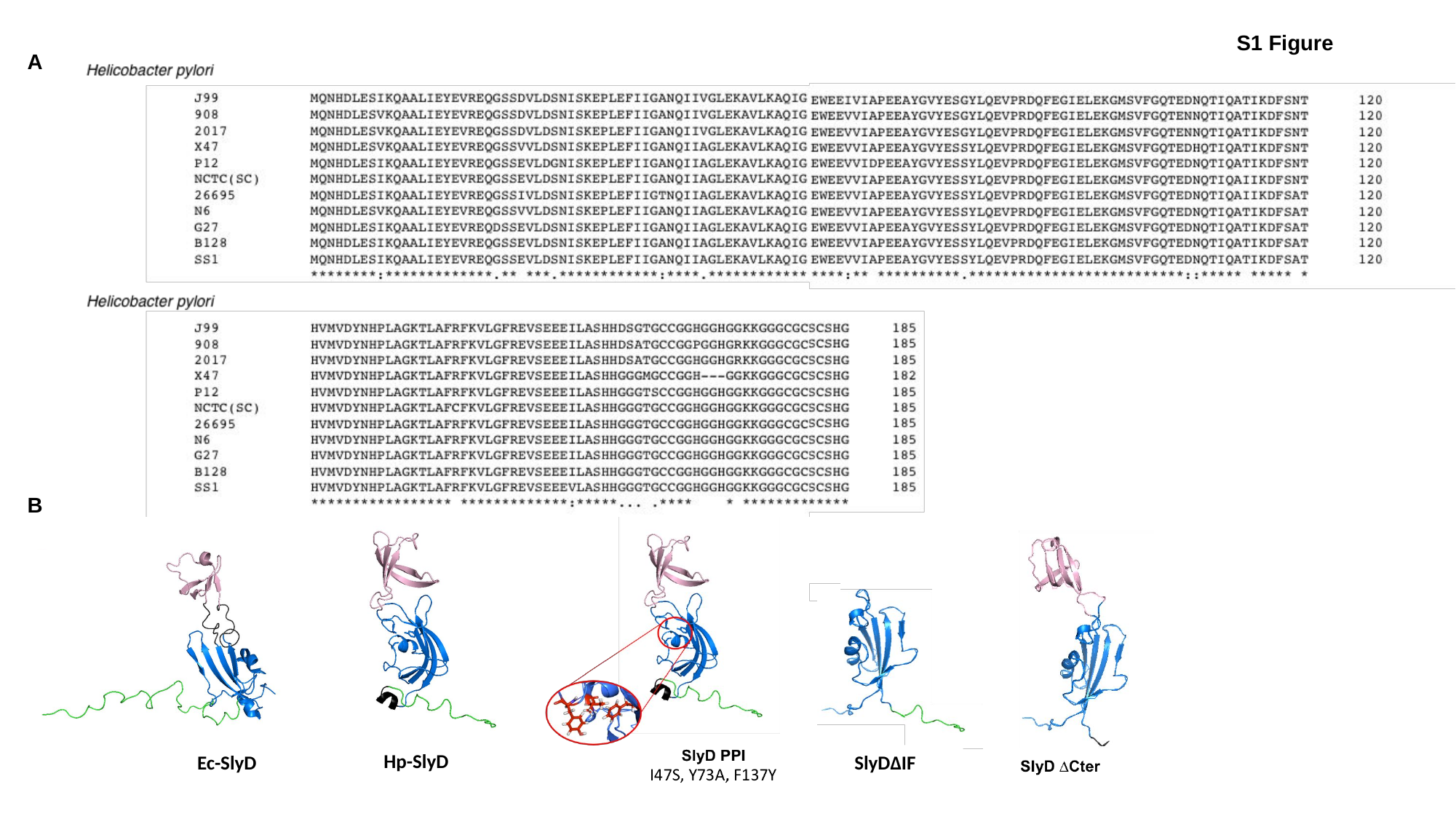

S1 Figure
A
B
Hp-SlyD
Ec-SlyD
SlyD∆IF

Supplement: S1 Fig — A. The alignment of the aminoacid sequence of a selection of SlyD proteins from 10 different H. pylori strains highlights a strong sequence conservation. Protein sequences were extracted from Kegg database and multiple alignment was made with Clustal Omega (https://www.ebi.ac.uk/Tools/msa/clustalo/). B. Prediction of the tridimensional structures of H. pylori mutant SlyD proteins. The NMR structure of E. coli SlyDΔCter protein (2KFW PDB [49]) served to represent its structure. When relevant, the C-terminal metal-binding region of unknown structure has been added for clarity (in green). The NMR structure of H. pylori SlyDΔCter protein [2KFW PDB [24]] served to represent its structure. Structure models of SlyD mutants was obtained with the Phyre2 software and modelized using PyMol. The PPIase domain is represented in blue, chaperone IF domain in pink and C-terminal region in green. (PPTX) [file ppat.1009193.s001.pptx]

## Slide 1
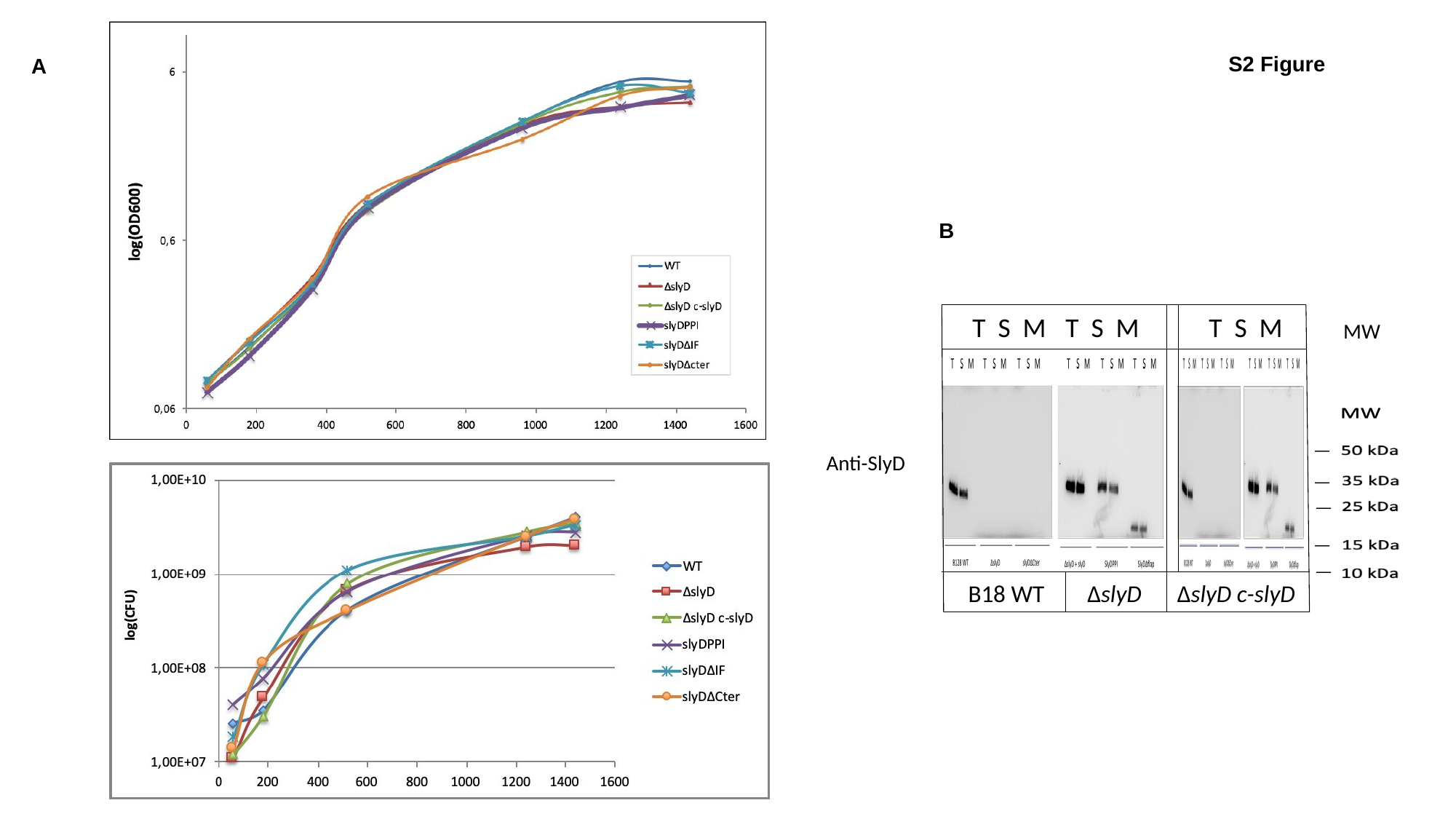

S2 Figure
A
B
T S M
T S M
T S M
MW
Anti-SlyD
B18 WT
∆slyD
∆slyD c-slyD

Supplement: S2 Fig — A. Growth curve and colony forming units (CFU) counts of WT and slyD mutant strains grown in BB medium at 37°C and followed during 1,500 min. Growth was followed by measuring OD600 and viability by CFU counting. The graphics represent a mean of three independent experiments and show that slyD deletion or mutations does not significantly impact H. pylori growth. B. Analysis of the subcellular localization of the SlyD protein in H. pylori by fractionation. Western blot was probed with anti-SlyD polyclonal antibodies on total extracts (T), soluble extract (S) and inner membrane (M) fractions prepared from H. pylori B128 wild type strain, a ΔslyD mutant and a ΔslyD-c slyD complemented strain. SlyD is exclusively detected in the soluble fraction, testifying of its cytosolic localization. (PPTX) [file ppat.1009193.s002.pptx]

## Slide 1
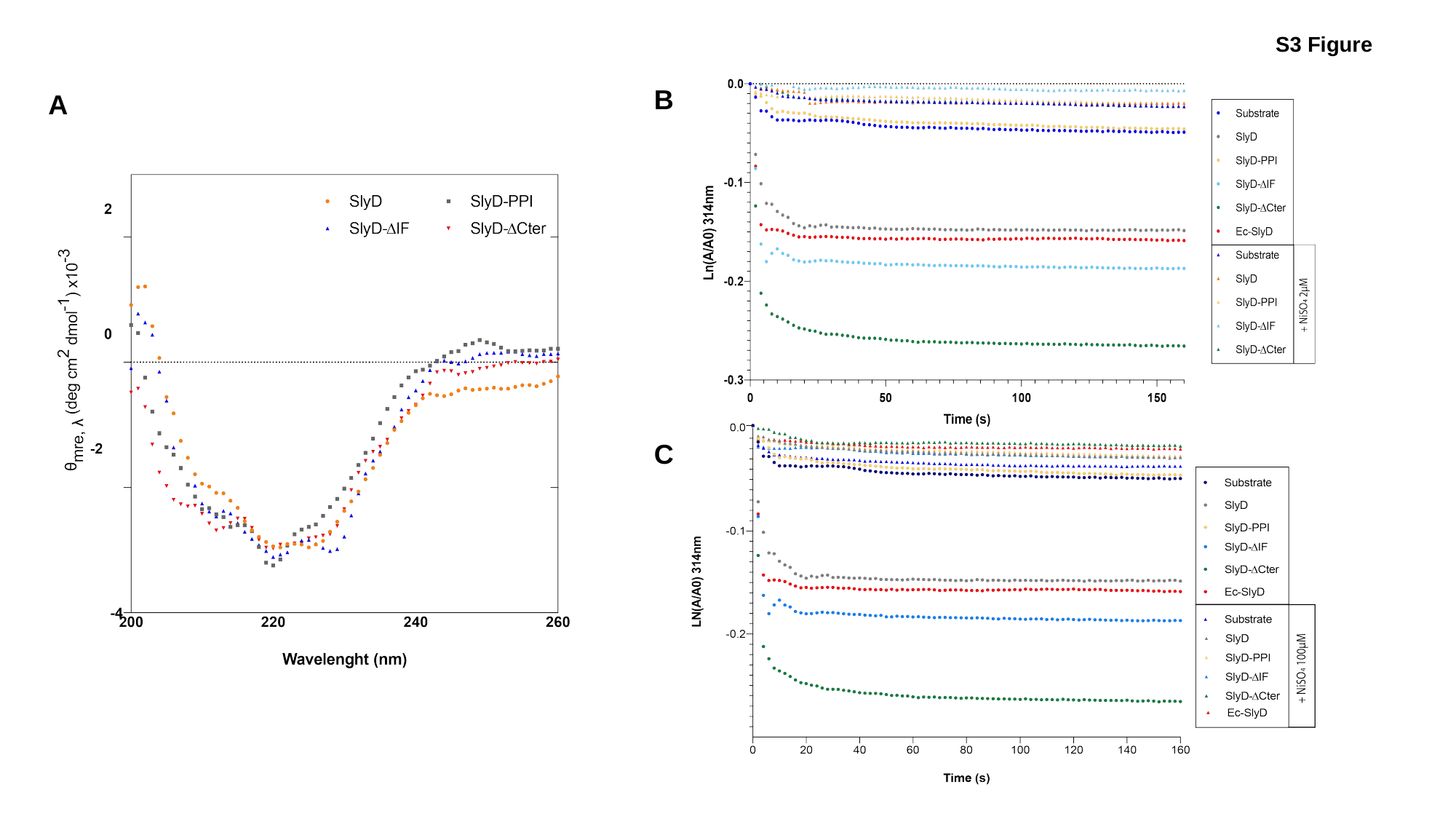

S3 Figure
B
A
C

Supplement: S3 Fig — A. Circular Dichroism spectra of SlyD wild type and mutant proteins were analyzed in 5 mM phosphate buffer (pH = 7.6). Five scans were averaged for each sample. B and C. A protease-free assay was used to measure PPIase activity of purified WT and mutant SlyD proteins without nickel or after addition of NiSO4 at 2 μM (panel B) or 100 μM (panel C). PPIase activity of purified E. coli SlyD was measured as a control. The time course of the reversible cis to trans first-order prolyl isomerization of a tetrapeptide substrate was followed by the decrease of absorbance recorded at 314 nm and represented as ln(A/A0). The average of three independent experiments is represented. (PPTX) [file ppat.1009193.s003.pptx]

## Slide 1
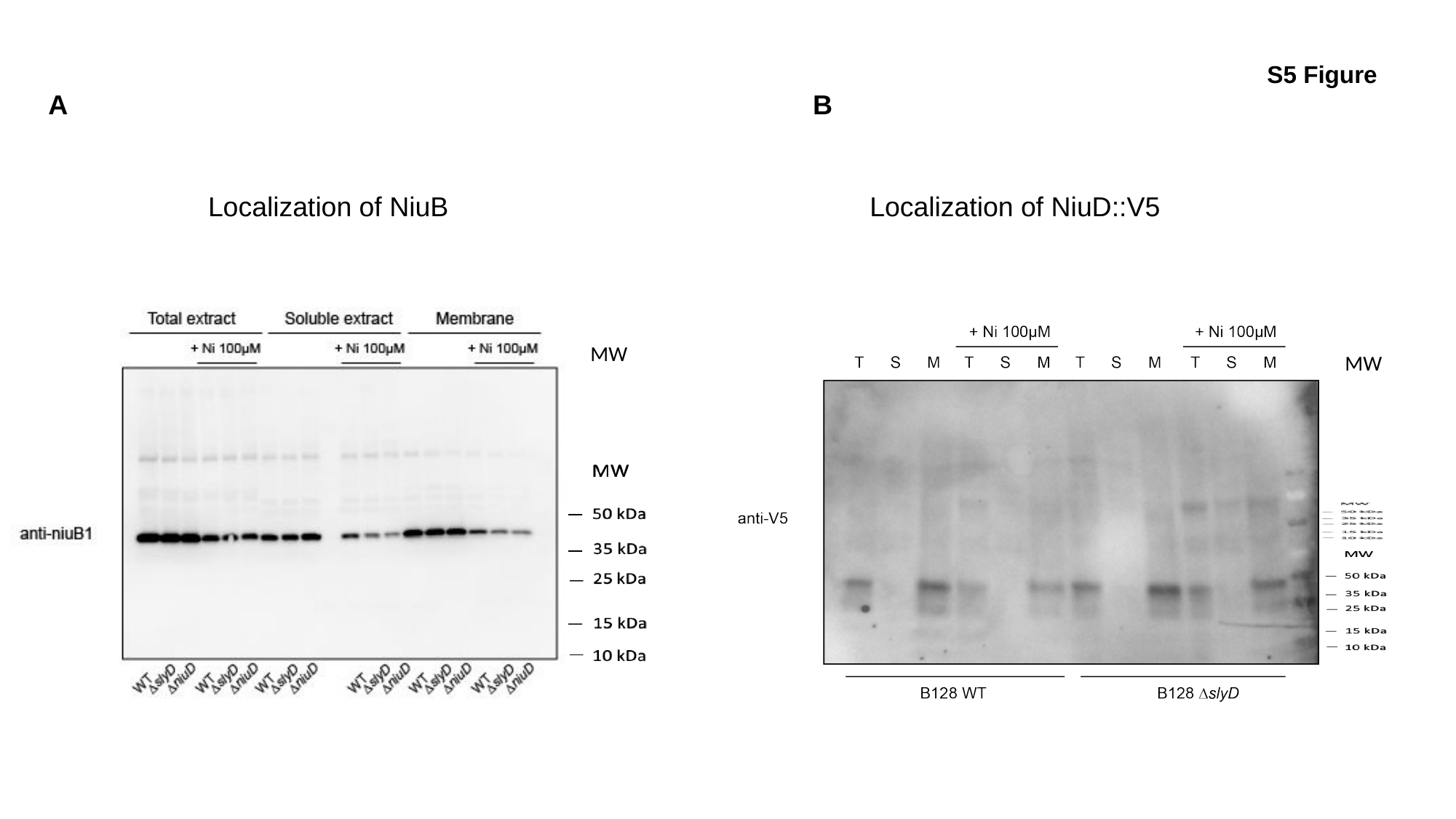

S5 Figure
A
B
Localization of NiuB
Localization of NiuD::V5
MW
MW

Supplement: S5 Fig — Western blot analysis of total extracts (T), soluble extract (S) and inner membrane (M) fractions prepared from H. pylori B128 wild type strain and ΔslyD mutant, each expressing NiuD-V5 fusion, grown without or with 100 μM of NiCl2. Western blots were revealed with anti-NiuB (panel A) and anti-V5 antibodies (panel B). The amount of both NiuD and NiuB1 proteins was diminished in the presence of nickel as expected. NiuB protein (35 kDa) was found in both the soluble (that comprises the periplasmic fraction) and inner membrane fractions in similar amounts in the WT, ΔslyD and ΔniuD strains. NiuD-V5 protein (35 kDa) is exclusively localized in the inner membrane in similar amounts of WT strain and ΔslyD mutant. (PPTX) [file ppat.1009193.s005.pptx]

## Slide 1
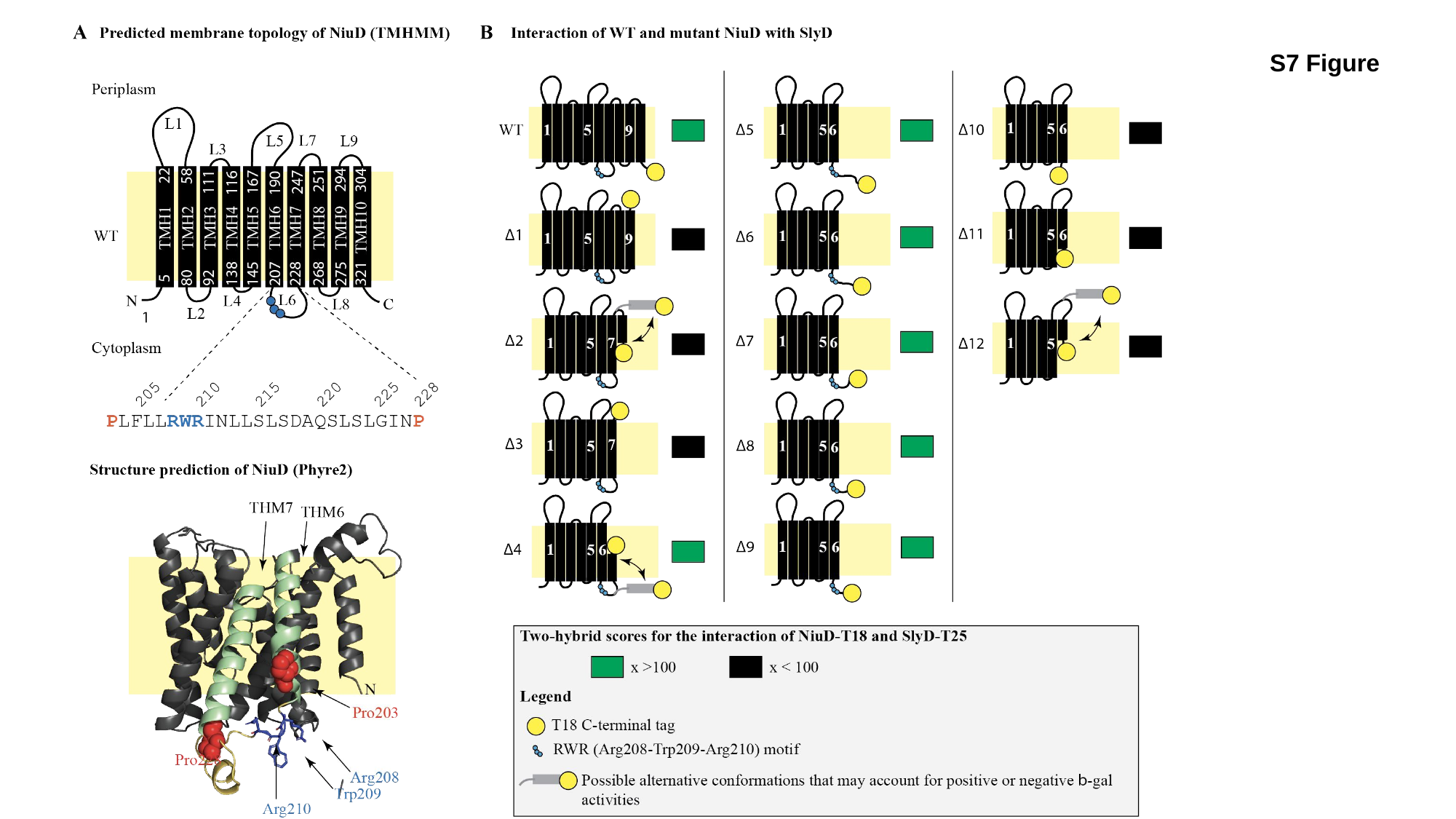

S7 Figure

Supplement: S7 Fig — A. Schematic representation of the predicated topology of a NiuD monomer as determined by the TMHMM program (Upper panel). N denotes the N-terminus, C the C-terminus of the protein; cylinders represent the 10 transmembrane helices (TMH) of NiuD and L1-L9 the periplasmic (L1, 3, 5, 7 and 9) and cytoplasmic (L2, 4, 6 and 8) loops (L) between TMHs. First and last residues flanking TMHs are indicated (numbering). L6’s sequence is indicated with the two proline residues in red and the RWR motif highlighted in blue; the RWR residues are depicted by blue circles on the scheme. Lower panel shows the structure prediction of a NiuD monomer (Phyre2 program, cartoon representation). TMH6 and 7 are green and L6 is orange, with the RWR motif (residues represented with blue sticks), and the two proline residues (spheres) flanking the loop in red. B. Schematic representation of NiuD’s and NiuD’s mutants (Δ1 to Δ12) topology. Each mutant’s name is indicated on the left and numbers indicate TMHs. The yellow circle depicts the T18 two-hybrid tag. In some cases, this tag might adopt alternative orientations/topologies that are indicated with grey cartoons. The periplasm is located above each figure, while the cytoplasm is located below. When T18 lies in or orientates towards the periplasmic side, it cannot participate to the interaction with SlyD-T25 that is located in the cytoplasm. Results of two-hybrid assays (SlyD-T25 and NiuD-T18 interactions) are indicated in each case with a color code. (PPTX) [file ppat.1009193.s007.pptx]

## Slide 1
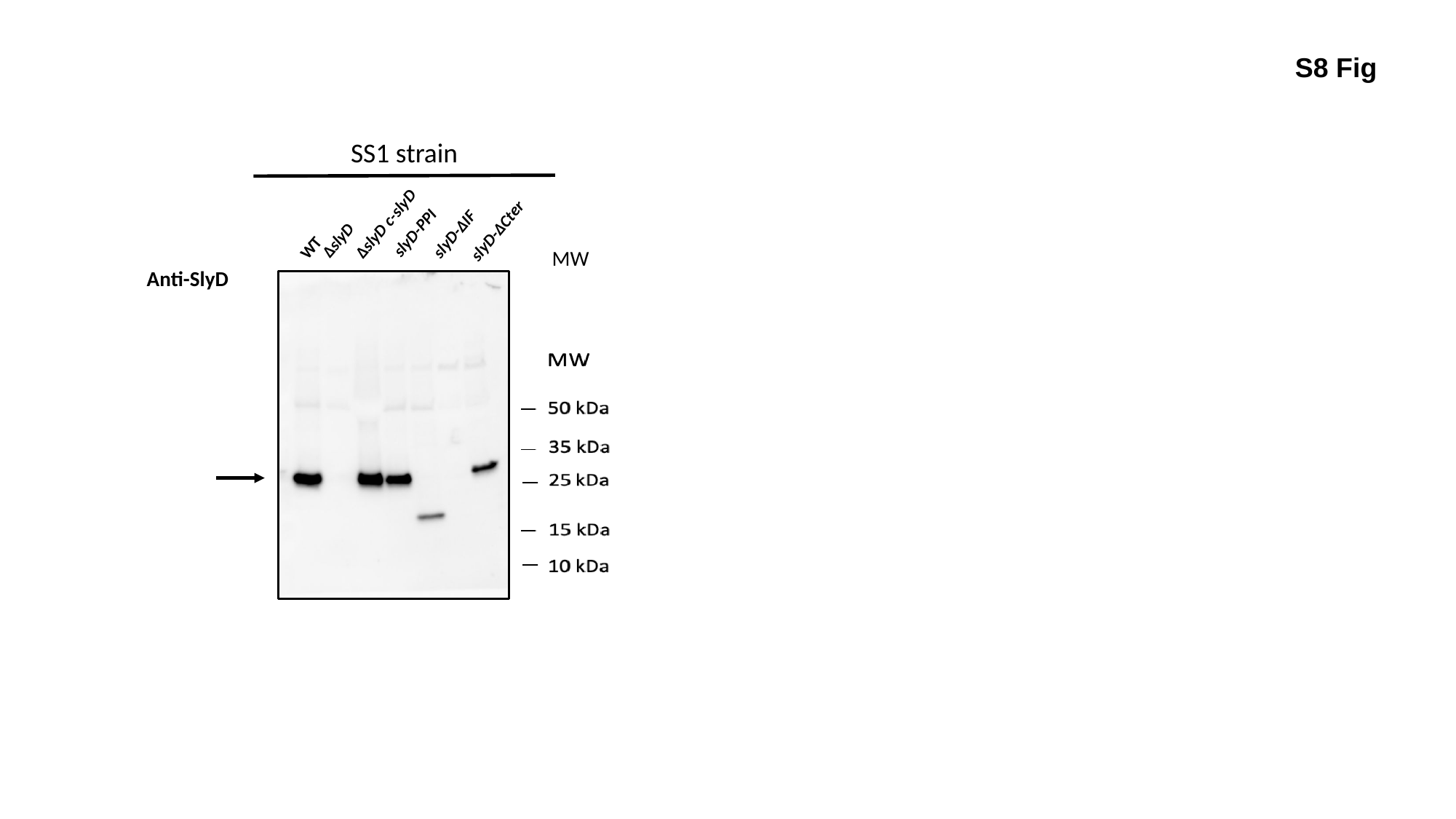

S8 Fig
SS1 strain
slyD-PPI
slyD-∆IF
∆slyD c-slyD
slyD-∆Cter
∆slyD
WT
MW
Anti-SlyD

Supplement: S8 Fig — H. pylori SS1 WT strain and SS1-derived mutants carrying the following mutations ΔslyD, ΔslyD c-slyD (complemented strain), slyD-PPI, slyD-ΔIF, and slyD-ΔCter strain, that were probed with specific anti-SlyD polyclonal antibodies prepared during this study. (PPTX) [file ppat.1009193.s008.pptx]

## Slide 1
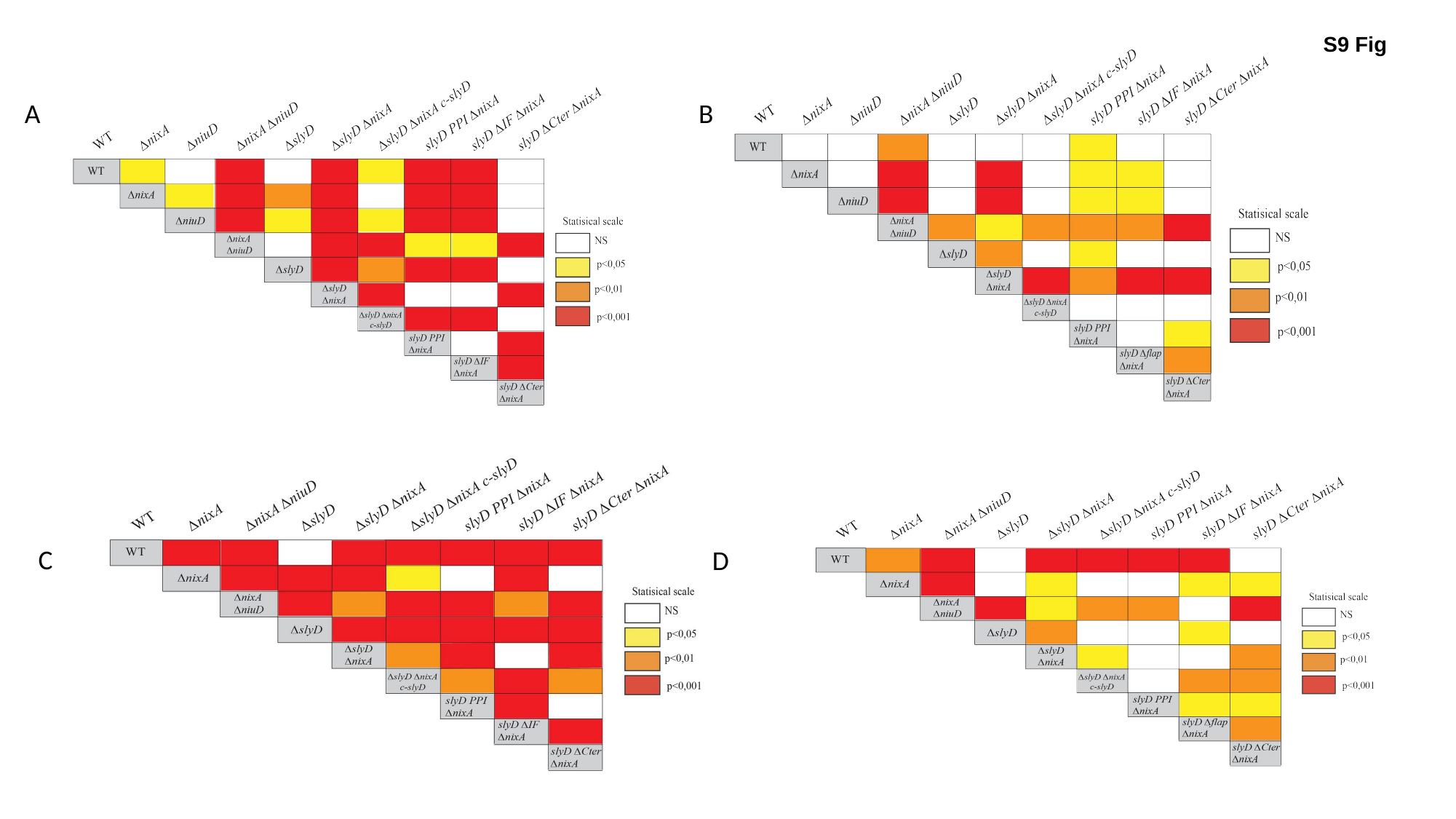

S9 Fig
A
B
C
D

Supplement: S9 Fig — The Student's t-test was used to determine significant differences between every pair of mean values from Figs 4, 5, 6 and 7. A color code indicates the p values for each comparison. A. Statistical analysis of the values of tolerance to toxic nickel exposure of H. pylori wild type and mutants from Fig 4. B. Statistical analysis of the values of the nickel content reporter of H. pylori wild type and mutants from Fig 5. C. Statistical analysis of the values of the ICP-OES nickel content measurements of H. pylori wild type and mutants from Fig 6. D. Statistical analysis of the values of radioactive nickel uptake rates of H. pylori wild type and mutants from Fig 7. (PPTX) [file ppat.1009193.s009.pptx]
