# Supplementary material for: A novel mode of control of nickel uptake by a multifunctional metallochaperone
Source: PLoS Pathog. 2021 Jan 14;17(1):e1009193. doi: 10.1371/journal.ppat.1009193 (PMC7840056; doi:10.1371/journal.ppat.1009193)
Supplement: S4 Fig — qRT-PCR normalized fold changes of the expression of niuD and niuB1 genes in a B128 wild type strain and ΔslyD mutant. ppK was used as the housekeeping gene for normalization of the values. These results are the means with the standard deviations of two independent experiments. (PPTX) [file ppat.1009193.s004.pptx]

## Slide 1
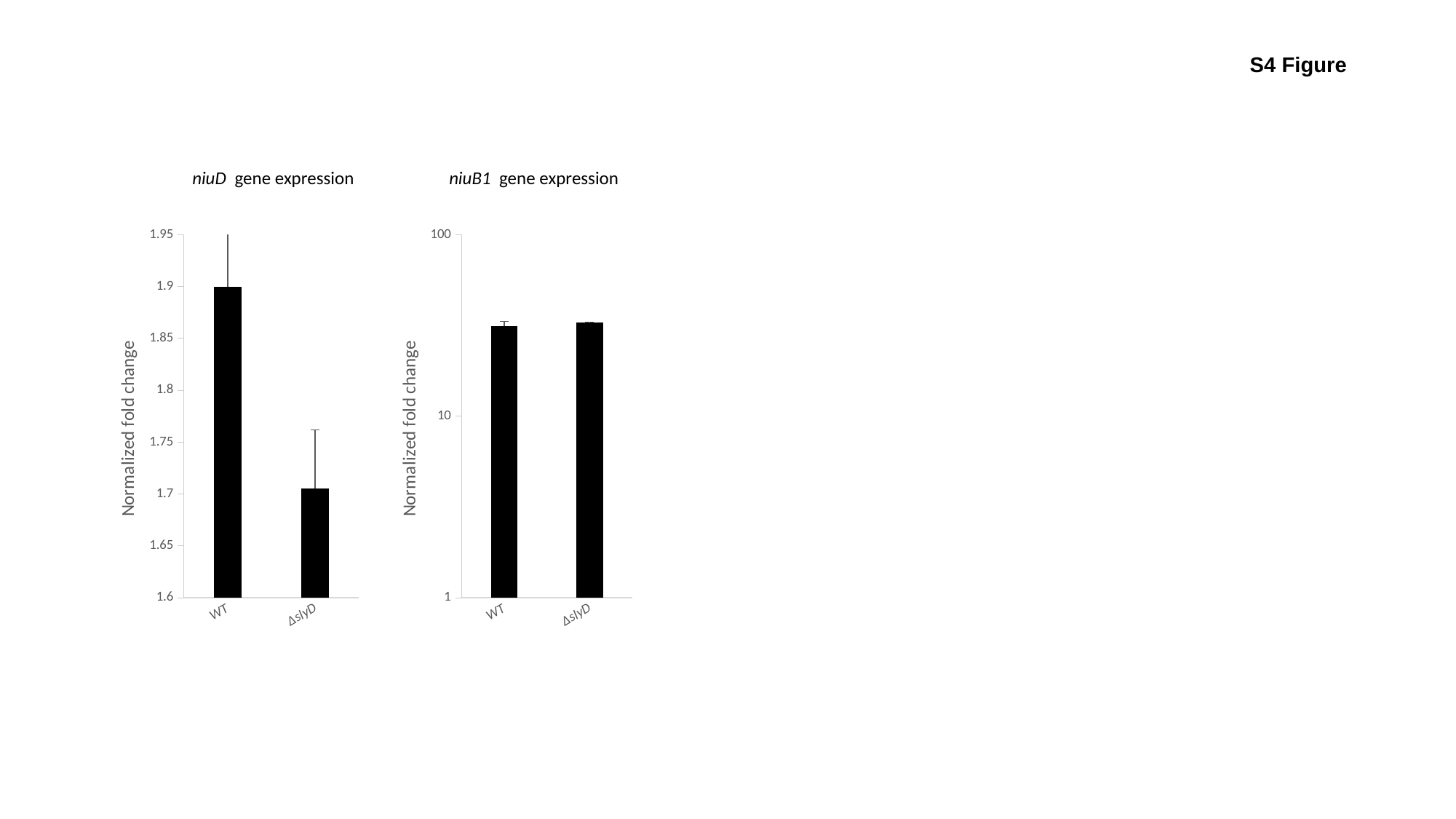

S4 Figure
niuD gene expression
niuB1 gene expression
### Chart
| Category | |
|---|---|
| WT | 1.9 |
| ∆slyD | 1.705 |
### Chart
| Category | |
|---|---|
| WT | 31.240000000000002 |
| ∆slyD | 32.84 |
