# Supplementary material for: A novel mode of control of nickel uptake by a multifunctional metallochaperone
Source: PLoS Pathog. 2021 Jan 14;17(1):e1009193. doi: 10.1371/journal.ppat.1009193 (PMC7840056; doi:10.1371/journal.ppat.1009193)
Supplement: S6 Fig — Western blot analysis of total extracts (T), soluble extract (SE) and inner membrane (IM) fractions prepared from E. coli BTH101 co-transformed with pU18::niuDΔ5 and pNKT25::slyD or pNKT25::slyD-PPI. Western blot was revealed with anti-Cya antibodies. NiuDΔ5 fused to the T18 Cya fragment is located in the E. coli inner membrane. B. Western blot analysis of total extracts prepared from E. coli BTH101 carrying SlyD wild type and mutant proteins fused to the T25 fragment and revealed with anti-SlyD antibodies. The production of SlyD-ΔCter-T25 fusion is sharply diminished in comparison with WT SlyD-T25 fusion protein. (PPTX) [file ppat.1009193.s006.pptx]

## Slide 1
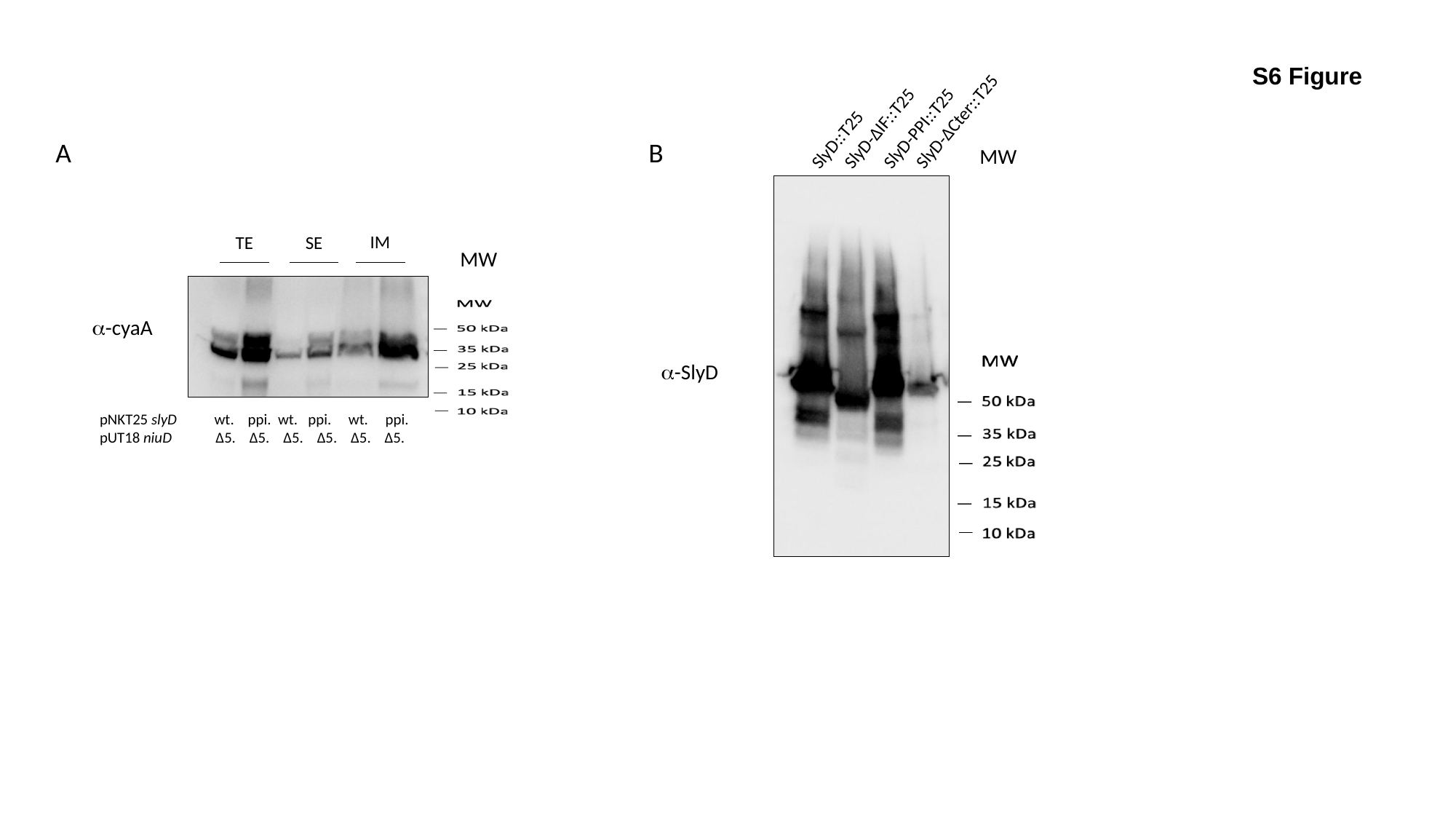

S6 Figure
SlyD-∆Cter::T25
SlyD-∆IF::T25
SlyD-PPI::T25
SlyD::T25
A
B
MW
IM
SE
TE
MW
α-cyaA
α-SlyD
pNKT25 slyD wt. ppi. wt. ppi. wt. ppi.
pUT18 niuD 	 ∆5. ∆5. ∆5. ∆5. ∆5. ∆5.
