# Supplementary material for: A novel mode of control of nickel uptake by a multifunctional metallochaperone
Source: PLoS Pathog. 2021 Jan 14;17(1):e1009193. doi: 10.1371/journal.ppat.1009193 (PMC7840056; doi:10.1371/journal.ppat.1009193)
Supplement: S1 Table — (DOCX) [file ppat.1009193.s010.docx]

**Table S1: Urease et hydrogenase activities of the WT B128 strain and its isogenic *∆slyD* mutant.**

|  | Urease activity  (U) | Hydrogenase activity  (U) |
| --- | --- | --- |
| B128 WT | 4.84 (± 0,11) | 8.3 (± 0,37) |
| B128 ∆*slyD* | 5.99 (± 0,63) | 8.7 (± 2,36) |

- One unit (U) of urease activity was defined as the amount of enzyme that generates 1 μmol ammonia per min per mg of total proteins.

- One unit (U) of hydrogenase activity was defined as the amount of enzyme that catalyzes the oxidation of 1 nmol H2 per min per mg of total proteins.
