# Supplementary material for: A novel mode of control of nickel uptake by a multifunctional metallochaperone
Source: PLoS Pathog. 2021 Jan 14;17(1):e1009193. doi: 10.1371/journal.ppat.1009193 (PMC7840056; doi:10.1371/journal.ppat.1009193)
Supplement: S2 Table — (DOCX) [file ppat.1009193.s011.docx]

**Table S2: strains used in this study**

| **Strains** | **Relevant characteristics** | **Reference** |
| --- | --- | --- |
| *Escherichia coli* | | |
| BTH101 | *F^-^, cya-99, araD139, galE15, galK16, rpsL1 (Str^R^), hsdR2, mcrA1, mcrB1* | (1) |
| XL1-Blue | *recA1 endA1 gyrA96 thi-1 hsdR17 supE44 relA1 lac* [F*´ proAB lacI^q^Z ΔM15* Tn10 (TetR)] | Agilent Technologies |
| BL21(DE3) *∆slyD::apra* | Fdcm ompT hsdS(rBmB- ) gal λ(DE3), Apra^R^ | This work |
| BL21(DE3) *∆slyD::apra* *p*ET28*::slyD* | Strain for SlyD overexpression used for *in vitro* assay and antibody synthesis, Apra^R^, Km^R^ | This work |
| BL21(DE3) *∆slyD::apra* *p*ET28*::slyD-PPI* | Strain for SlyD-PPI overexpression used for *in vitro* assay, Apra^R^, Km^R^ | This work |
| BL21(DE3) *∆slyD::apra* *p*ET28*::slyD-∆IF* | Strain for SlyD-∆IF overexpression used for *in vitro* assay, Apra^R^, Km^R^ | This work |
| BL21(DE3) *∆slyD::apra* *p*ET28*::slyD-∆Cter* | Strain for SlyD-∆Cter overexpression used for *in vitro* assay, Apra^R^, Km^R^ | This work |
| BL21(DE3) *∆slyD::apra* *+ p*ET15(MHL)*::niuB1* | Strain for NiuB1 overexpression used for antibody synthesis, Apra^R^, Amp^R^ | This work |
| *Helicobacter pylori* | | |
| B128 | Reference strain | (2, 3) |
| B128 ∆*niuD* | Unmarked deletion mutant, Strepto^R^ | (4) |
| B128 ∆*nixA*::*km* | Km^R^, Strepto^R^ | (4) |
| B128 ∆*nixA*::*km* ∆*niuD* | Unmarked *niuD* deletion mutant, Km^R^, Strepto^R^ | (4) |
| B128 ∆*slyD* | Unmarked *SlyD* deletion mutant, Strepto^R^ | This work |
| B128 *∆slyD c-slyD* | Unmarked *slyD* deletion mutant, Strepto^R^, *slyD* at the chromosomal locus Apra^R^ | This work |
| B128 *slyD-PPI* | *slyD-PPI* point mutations on the chromosomal locus Apra^R^, Strepto^R^ | This work |
| B128 *slyD-∆IF* | *slyD-∆IF* deletion at the chromosomal locus Apra^R^, Strepto^R^ | This work |
| B128 *slyD-Cter* | *slyD-∆Cter* [1-155] deletion at the chromosomal locus Apra^R^, Strepto^R^ | This work |
| B128 *∆slyD ∆nixA::km* | Unmarked *slyD* deletion mutant, Km^R^, Strepto^R^ | This work |
| B128 *∆slyD ∆nixA::km c-slyD* | Unmarked *slyD* deletion mutant, Km^R^, Strepto^R^, recomplemented at the chromosomal locus | This work |
| B128 *slyD-PPI ∆nixA::km* | *slyD-PPI* point mutations at the chromosomal locus Apra^R^, Km^R^, Strepro^R^ | This work |
| B128 *slyD-∆IF ∆nixA::km* | *slyD-∆IF* deletion on the chromosome locus Apra^R^, Km^R^, Strepro^R^ | This work |
| B12 *slyD-Cter ∆nixA::Km* | *slyD-∆Cter* deletion on the chromosome locus Apra^R^, Km^R^, Strepro^R^ | This work |
| B128 *niuD-V5::km* | B128 Strepto^R^, *niuD* fused with a V5 tag Km^R^ | This work |
| B128 *∆slyD niuD-V5::km* | B128 Strepto^R^, unmarked *slyD* deletion mutant, *niuD* fused with a V5 tag Km^R^ | This work |
| B128 *∆czn* | *∆cznABC*, Apra^R^, Strepto^R^ | This work |
| B128 *∆czn ∆slyD* | *∆cznABC*, Apra^R^, Strepto^R^, unmarked *slyD* deletion mutant | This work |
|  | | |
|  | | |
| **SS1** | Sequenced parental strain | (4, 5) |
| SS1 *∆slyD c-slyD* | Unmarked deletion mutant, Strepto^R^, *slyD* on chromosome locus Apra^R^ | This work |
| SS1 *slyD-PPI* | *slyD-PPI* point mutations on the chromosome locus Apra^R^, Strepto^R^ | This work |
| SS1 *slyD-∆IF* | *slyD-∆IF* deletion on the chromosome locus Apra^R^, Strepto^R^ | This work |
| SS1 *slyD ∆Cter* | *slyD-∆Cter* deletion on the chromosome locus Apra^R^, Strepto^R^ | This work |

**References**

1. G. Karimova, A. Ullmann, D. Ladant, A bacterial two-hybrid system that exploits a cAMP signaling cascade in *Escherichia coli*. *Methods Enzymol.* **328**, 59–73 (2000).

2. M. S. McClain, C. L. Shaffer, D. A. Israel, R. M. Peek, T. L. Cover, Genome sequence analysis of *Helicobacter pylori* strains associated with gastric ulceration and gastric cancer. *BMC Genomics* **10**, 1–14 (2009).

3. M. Farnbacher, *et al.*, Sequencing, annotation, and comparative genome analysis of the gerbil-adapted *Helicobacter pylori* strain B8. *BMC Genomics* **11** (2010).

4. F. Fischer, *et al.*, Characterization in *Helicobacter pylori* of a nickel transporter essential for colonization that was acquired during evolution by gastric *Helicobacter* species. *PLoS Pathog.* **12**, 1–31 (2016).

5. A. Lee, *et al.*, A standardized mouse model of *Helicobacter pylori* infection: Introducing the Sydney strain. *Gastroenterology* **112**, 1386–1397 (1997).
