# Supplementary material for: A novel mode of control of nickel uptake by a multifunctional metallochaperone
Source: PLoS Pathog. 2021 Jan 14;17(1):e1009193. doi: 10.1371/journal.ppat.1009193 (PMC7840056; doi:10.1371/journal.ppat.1009193)
Supplement: S3 Table — (DOCX) [file ppat.1009193.s012.docx]

**Table S3: plasmids used in this study**

| **Plasmids** | **Relevant characteristics** | **Reference** |
| --- | --- | --- |
| *Vectors* | | |
| *p*GEM-T | Cloning vector | Novagen |
| *p*NKT25 | BACTH vector designed to express a protein fused in frame at its C-terminus with T25 domain of CyaA; p15 ori; Km^R^ | (1) |
| *p*UT18 | BACTH vector designed to express a protein fused in frame at its C-terminus with T18 domain of CyaA; ColE1 ori; Amp^R^ | (1) |
| *p*ET28(a)+ | N-terminal His-Tag/thrombin/T7-Tag and an optional C-terminal His-Tag sequence, Km^R^ | Novagen |
| *Plasmids* | | |
| *pILL2157 (*P*fecA3::lacZ)* | Plasmid expressing the P*fecA3::lacZ* fusion regulated by NikR into pILL2157, Cm^R^ | (2) |
| *p*ET28*::slyD*  *p*ET28*::slyD-PPI*  *p*ET28*::slyD-∆IF*  *p*ET28*::slyD-∆Cter* | Plasmids for production of WT and mutant *slyD* proteins | This work |
| *p*ET15(MHL)*::niuB1* | Plasmids for *niuB1* overexpression, Amp^R^ | gift from C. Calmettes (INRS) |
| *p*NKT25*::slyD*  *p*NKT25*::slyD-PPI*  *p*NKT25*::slyD-∆IF*  *p*NKT25*::slyD-∆Cter* | SlyD (WT) and mutants BACTH expression plasmids | This work |
| *p*UT18*::niuB1* | NiuB1 BACTH expression plasmid | This work |
| *p*UT18*::niuE* | NiuB1 BACTH expression plasmid | This work |
| *p*UT18*::niuD*  *p*UT18*::niuD∆1*  *p*UT18*::niuD∆2*  *p*UT18*::niuD∆3*  *p*UT18*::niuD∆4*  *p*UT18*::niuD∆5*  *p*UT18*::niuD∆6*  *p*UT18*::niuD∆7*  *p*UT18*::niuD∆8*  *p*UT18*::niuD∆9*  *p*UT18*::niuD∆10*  *p*UT18*::niuD∆11*  *p*UT18*::niuD∆12* | NiuD full length and truncated BACTH expression plasmids | This work |
| *p*UT18 ::(NiuD∆7-P203V)  *p*UT18 ::(NiuD∆7-R208D)  *p*UT18 ::(NiuD∆7-R208G)  *p*UT18 ::(NiuD∆7-W209F)  *p*UT18 ::(NiuD∆7-W209G)  *p*UT18 ::(NiuD∆7-R210G)  *p*UT18::(NiuD∆7-RWR-GGG) | NiuD∆7-point mutation BACTH expression plasmids | This work |

**References**

1. G. Karimova, A. Ullmann, D. Ladant, A bacterial two-hybrid system that exploits a cAMP signaling cascade in *Escherichia coli.* *Methods Enzymol.* **328**, 59–73 (2000).

2. F. Fischer, *et al.*, Characterization in *Helicobacter pylori* of a nickel transporter essential for colonization that was acquired during evolution by gastric *Helicobacter* Species. *PLoS Pathog.* **12**, 1–31 (2016).
