# Supplementary material for: A novel mode of control of nickel uptake by a multifunctional metallochaperone
Source: PLoS Pathog. 2021 Jan 14;17(1):e1009193. doi: 10.1371/journal.ppat.1009193 (PMC7840056; doi:10.1371/journal.ppat.1009193)
Supplement: S4 Table — (DOCX) [file ppat.1009193.s013.docx]

**S4 Table: oligonucleotides used in this study**

| **Primer** | **Sequence (5’-3’)** |
| --- | --- |
| **Construction of the *slyD* unmarked mutant** | |
| *slyD-flankA1* | Tccaaaaagccctcaaagaa |
| *slyD-flankA2* | aaactcgagggtggtgtttctccttgttttc |
| *slyD-flankB1* | aaaggatcctaaggtataggagtctttaa |
| *slyD-flankB2* | gcaagaataaaccccctag |
| *difHrpsLcat-1* | ccgCTCGAGATTTAAAAGTTTGAAAAG |
| *difHrpsLcat-2* | CGCGGATCCATCGATCATTTAGTTATG |
| **Construction of *the ∆slyD c-slyD, slyD-PPI, slyD-∆IF* and *slyD-∆Cter,* Apra^R^** | |
| *slyD-apra* | CCCCTTTTGCTGATGGAGCTGCACATGAACCCCTACCCATGCGAACATGAGCAACCACAACC |
| *slyD-∆cter-apra* | CTGGGGCAACACGTGGAGCGGATCGTAAGGTATAGGAGTCTTTAAAAGGCAAGG |
| *Apra-FD* | gggttcatgtgcagctcca |
| *Apra-RV* | ggcaacacgtggagcggatcg |
| **Construction of *niuD-V5::Km*** | |
| *niuD-FD* | AAATTGAGCGCGTTTTTAAGCGCC |
| *niuD-RV* | AAATTCTGGCTAAAAGCACCATTTGCC |
| **Construction of the *slyD, slyD-PPI, slyD-∆IF* and *slyD-∆Cter* in the pET28 vector** | |
| *slyD FD* | AATTCCATatgCAAAACCATGATTTAGAAT |
| *slyD RV* | ccgctcgagctaCCCATGCGAACATGAGC |
| *slyD-∆Cter RV* | CCGGAATTCCGGTGAAGCTAAAATTTCTTCTTCGC |
| **Constructions for the bacterial two hybrid screen** | |
| Cloning of *slyD, slyD-PPI, slyD-∆IF* and *slyD-∆Cter* into pNKT25 | |
| *slyD-NKT25 FD* | aaactgcagggATGcaaaaccatgatttagaatc |
| *slyD-NKT25 RV* | aaagaattcgaactattcatgaccttgcc |
| *slyD-∆Cter-NKT25 RV* | CCGGAATTCGGTGAAGCTAAAATTTCTTCTTCGC |
| Cloning of *niuB1* into pUT18 | |
| *niuB1-18 FV* | AACTGCAGatgctaatcgctcgctttaaaaaagc |
| *niuB1-18 RV* | CGGAATTCcgatgccataagaaaggttcaac |
| Cloning of *niuE* into pUT18 | |
| *niuE-18 FV* | TACGCCAAGCTTGCATGCCTGCAGAAAAGGAAGCGTGatgGTCTTAGAAG  TTAAAAAC |
| *niuE-18 RV* | CCTCGCTGGCGGCTGAATTCAACGCATACACGACAAGCTTG |
| Cloning of *niuD* full length and truncated into pUT18 | |
| *niuD-18 FV* | TACGCCAAGCTTGCATGCCTGCAGAAAAAGGAAGCGTGatgCTTAAAACC  TATC |
| *niuD-18 RV* | CCTCGCTGGCGGCTgaattCACCCCCCTAGTTCTAAAC |
| *niuD∆1-18 RV* | CCTCGCTGGCGGCTGAATTcaccacatccgctaaaagcaag |
| *niuD∆2-18 RV* | CCTCGCTGGCGGCTGAATTCAACCCAATCCAGCCAATCGT |
| *niuD∆3-18 RV* | CCTCGCTGGCGGCTGAATTCACCGCTAAAGCGCTCGCAAC |
| *niuD∆4-18 RV* | CCTCGCTGGCGGCTGAATTCAGCGATCGCAACAACACCGG |
| *niuD∆5-18 RV* | CCTCGCTGGCGGCTGAATTCGGGTTAATCCCCAAACTCAA |
| *niuD∆6-18 RV* | CCTCGCTGGCGGCTGAATTCCCCAAACTCAAACTTTGTGC |
| *niuD∆7-18 RV* | CCTCGCTGGCGGCTGAATTCAAGCTCAATAAATTAATGCG |
| *niuD∆8-18 RV* | CCTCGCTGGCGGCTGAATTCAAATTAATGCGCCACCTTAA |
| *niuD∆9-18 RV* | CCTCGCTGGCGGCTGAATTCCGCCACCTTAACAAAAAAAG |
| *niuD∆10-18 RV* | CCTCGCTGGCGGCTGAATTCAACAAAAAAAGGGGGATAAA |
| *niuD∆11-18 RV* | CCTCGCTGGCGGCTGAATTCGGGATAAAGCCTAAGGATAG |
| *niuD∆12-18 RV* | CCTCGCTGGCGGCTGAATTccctatgaaagcgatcaagca |
| Construction of *niuD∆7*-point mutations into pU18 | |
| *niuD∆7 R203V FD* | GCTATCCTTAGGCTTTATCGTCCTTTTTTTGTTAAGGTGGCG |
| *niuD∆7 R203V RV* | CGCCACCTTAACAAAAAAAGGACGATAAAGCCTAAGGATAGC |
| *niuD∆7 R208D FD* | TTTTTGTTACAGTGGCGGATTAATTTATTG |
| *niuD∆7 R208D RV* | CAATAAATTAATCCGCCACTGTAACAAAAA |
| *niuD∆7 R208G FD* | CCCGCTTTTTTTGTTAGGGTGGCGCATTAATTTATTG |
| *niuD∆7 R208G RV* | CAATAAATTAATGCGCCACCCTAACAAAAAAAGCGGG |
| *niuD∆7 W209G FD* | CCCGCTTTTTTTGTTAAGGGGACGCATTAATTTATTGAGCTTG |
| *niuD∆7 W209G RV* | CAAGCTCAATAAATTAATGCGTCCCCTTAACAAAAAAAGCGGG |
| *niuD∆7 W209F FV* | CCCGCTTTTTTTGTTAAGGTTTCGCATTAATTTATTGAGCTTG |
| *niuD∆7 W209F RV* | CAAGCTCAATAAATTAATGCGAAACCTTAACAAAAAAAGCGGG |
| *niuD∆7 R210G FD* | CGCTTTTTTTGTTAAGGTGGGGAATTAATTTATTG |
| *niuD∆7 R210G RV* | CAATAAATTAATTCCCCACCTTAACAAAAAAAGCG |
| *niuD∆7 RWR-GGG FD* | CCCGCTTTTTTTGTTAGGAGGAGGCATTAATTTATTGAGC |
| *niuD∆7 RWR-GGG RV* | GCTCAATAAATTAATGCCTCCTCCTAACAAAAAAAGCGGG |
| **RT-qPCR primers** | |
| *ppK FD* | GCGTTAGTCGTTTATGGCGTTT |
| *ppK RV* | CGCTTAAAGGGTTGTAATTGCC |
| *niuD FD* | GCGATAGCGGTAGTGGAGTC |
| *niuD RV* | GAAAGCACCAACGAAAGGAC |
| *niuB1 FV* | CGCTTCCAATCAAGAAGTCC |
| *niuB1 RV* | ATCCCAAGTATGGAGCATGG |

FD: forward

RV: reverse
