# Supplementary material for: A novel mode of control of nickel uptake by a multifunctional metallochaperone
Source: PLoS Pathog. 2021 Jan 14;17(1):e1009193. doi: 10.1371/journal.ppat.1009193 (PMC7840056; doi:10.1371/journal.ppat.1009193)
Supplement: S5 Table — (DOCX) [file ppat.1009193.s014.docx]

**Table S5: ß-galactosidase activity, expressed in Miller units, by the P*fecA::lacZ* fusion in *H. pylori* wild type and mutants after 24 hours.**

| Strain | Miller units  mean value (No NiCl_2_) | Miller units  mean value (+100µM NiCl_2_) |
| --- | --- | --- |
| WT | 6429 | 1499 |
| *∆nixA* | 8294 | 1860 |
| *∆niuD* | 8261 | 1783 |
| *∆nixA ∆niuD* | 6193 | 4669 |
| *∆slyD* | 8456 | 1715 |
| *∆slyD ∆nixA* | 6102 | 3208 |
| *∆slyD ∆nixA c-slyD* | 7753 | 2189 |
| *slyD-PPI ∆nixA* | 6415 | 2564 |
| *slyD-∆IF ∆nixA* | 8434 | 2655 |
| *slyD-∆Cter ∆nixA* | 7687 | 2299 |
