# Supplementary material for: A novel mode of control of nickel uptake by a multifunctional metallochaperone
Source: PLoS Pathog. 2021 Jan 14;17(1):e1009193. doi: 10.1371/journal.ppat.1009193 (PMC7840056; doi:10.1371/journal.ppat.1009193)
Supplement: S6 Table — (DOCX) [file ppat.1009193.s015.docx]

**Table S6: ß-galactosidase activity, expressed in Miller units, of the two-hybrid assays.**

1. Cotransformation with *p*NKT15::*slyD* (WT)

| *p*UT18 | Miller Units mean value | Standard deviation |
| --- | --- | --- |
| empty | 40 | 4 |
| *niuB1* | 37 | 5 |
| *niuE* | 32 | 8 |
| *niuD* | 2408 | 194 |
| *niuD∆1* | 26 | 5 |
| *niuD∆2* | 33 | 2 |
| *niuD∆3* | 18 | 3 |
| *niuD∆4* | 1861 | 64 |
| *niuD∆5* | 1552 | 308 |
| *niuD∆6* | 1791 | 200 |
| *niuD∆7* | 2455 | 120 |
| *niuD∆8* | 575 | 91 |
| *niuD∆9* | 560 | 112 |
| *niuD∆10* | 15 | 2 |
| *niuD∆11* | 21 | 3 |
| *niuD∆12* | 20 | 1 |
| *niuD∆7* | 2455 | 120 |
| *niuD∆7P203V* | 1484 | 155 |
| *niuD∆7R208Q* | 103 | 5 |
| *niuD∆7R208G* | 1011 | 124 |
| *niuD∆7W209F* | 1186 | 148 |
| *niuD∆7W209G* | 720 | 71 |
| *niuD∆7R210G* | 124 | 39 |
| *niuD∆7RWR-GGG* | 35 | 7 |

1. Cotransformation with pNKT25 *slyD* (WT) and mutants

|  | Miller Units | pUT18 | | | |
| --- | --- | --- | --- | --- | --- |
|  |  | *niuD* | *niuD∆1* | *niuD∆7* | *niuD∆7 RWR-GGG* |
| pNKT25 | *slyD-∆Cter* | 216,35 | 18 | 139 | 19 |
|  | *slyD-∆IF* | 701,27 | 32,36 | 447 | 218 |
|  | *slyD-PPI* | 1738 | 27,67 | 2093 | 94 |
|  | *slyD* | 2408 | 18,6 | 2455 | 35 |
|  |  |  |  |  |  |
|  | Standard deviation | pUT18 | | | |
|  |  | *niuD* | *niuD∆1* | *niuD∆7* | *niuD RWR-GGG* |
| pNKT25 | *slyD-∆Cter* | 40 | 3 | 47 | 2 |
|  | *slyD-∆IF* | 274 | 3 | 129 | 27 |
|  | *slyD-PPI* | 388 | 2 | 381 | 42 |
|  | *slyD* | 194 | 5 | 120 | 9 |
